# Artificial Neural Networks and Experimental Analysis of the Resistance Spot Welding Parameters Effect on the Welded Joint Quality of AISI 304

**DOI:** 10.3390/ma17092167

**Published:** 2024-05-06

**Authors:** Marwan T. Mezher, Alejandro Pereira, Tomasz Trzepieciński, Jorge Acevedo

**Affiliations:** 1 Departamento de Deseño na Enxeñaría, Universidade de Vigo, 36310 Vigo, Spain; apereira@uvigo.es; 2 Institute of Applied Arts, Middle Technical University, Baghdad 10074, Iraq; 3 Department of Manufacturing Processes and Production Engineering, Rzeszow University of Technology, al. Powst. Warszawy 8, 35-959 Rzeszów, Poland; tomtrz@prz.edu.pl; 4 Departamento de Enxeñaría Electrónica, Universidade de Vigo, 36310 Vigo, Spain; acevedo@uvigo.es

**Keywords:** resistance spot welding, AISI 304, artificial neural network, shear force, nugget diameter, micro-hardness, unequal thicknesses

## Abstract

The automobile industry relies primarily on spot welding operations, particularly resistance spot welding (RSW). The performance and durability of the resistance spot-welded joints are significantly impacted by the welding quality outputs, such as the shear force, nugget diameter, failure mode, and the hardness of the welded joints. In light of this, the present study sought to determine how the aforementioned welding quality outputs of 0.5 and 1 mm thick austenitic stainless steel AISI 304 were affected by RSW parameters, such as welding current, welding time, pressure, holding time, squeezing time, and pulse welding. In order to guarantee precise evaluation and experimental analysis, it is essential that they are supported by a numerical model using an intelligent model. The primary objective of this research is to develop and enhance an intelligent model employing artificial neural network (ANN) models. This model aims to provide deeper knowledge of how the RSW parameters affect the quality of optimum joint behavior. The proposed neural network (NN) models were executed using different ANN structures with various training and transfer functions based on the feedforward backpropagation approach to find the optimal model. The performance of the ANN models was evaluated in accordance with validation metrics, like the mean squared error (MSE) and correlation coefficient (R^2^). Assessing the experimental findings revealed the maximum shear force and nugget diameter emerged to be 8.6 kN and 5.4 mm for the case of 1–1 mm, 3.298 kN and 4.1 mm for the case of 0.5–0.5 mm, and 4.031 kN and 4.9 mm for the case of 0.5–1 mm. Based on the results of the Pareto charts generated by the Minitab program, the most important parameter for the 1–1 mm case was the welding current; for the 0.5–0.5 mm case, it was pulse welding; and for the 0.5–1 mm case, it was holding time. When looking at the hardness results, it is clear that the nugget zone is much higher than the heat-affected zone (HZ) and base metal (BM) in all three cases. The ANN models showed that the one-output shear force model gave the best prediction, relating to the highest R and the lowest MSE compared to the one-output nugget diameter model and two-output structure. However, the Levenberg–Marquardt backpropagation (Trainlm) training function with the log sigmoid transfer function recorded the best prediction results of both ANN structures.

## 1. Introduction

The resistance spot welding (RSW) process is widely acknowledged as one of the oldest electric welding techniques in use today, and it is employed to join virtually all common metals. The welding operation is accomplished through the interaction of time, heat, and pressure [1].

Since the sheet metal to be connected has an electrical resistance to the current flow that is applied during the RSW process, it generates localized heating in a limited area at the interface of the sheet metals to be welded. It is recommended to use the RSW procedure instead of using mechanical fasteners, such as screws and rivets, when disassembling for maintenance is not requested [2]. The utilization of stainless steel grades in the manufacture of car components for the upcoming generation has been investigated due to its better features, including corrosion resistance and impact absorption capacity, in contrast to other mild steels [3,4,5].

Metro cars and intercity express trains are essential for the transportation infrastructure of contemporary urban cities. Austenitic stainless steel (ASS) is extensively used in the fabrication of metro vehicle components because of its affordability, strong properties, high resistance to corrosion, and great weldability in the RSW process [6,7,8,9,10]. However, unlike mild steel and aluminum alloys, austenitic stainless steel (ASS) has a high thermal expansion coefficient, making the conventional arc welding method unsuitable for welding ASS. Accordingly, the RSW process has been considered the most suitable welding method for manufacturing the ASS metro vehicle components [11,12].

According to [13], the microstructure of AISI 304 consisted of the austenite phase and a small amount of the ferrite phase. Gas metal arc welding (GMAW), gas tungsten arc welding (GTAW), and shielded metal arc welding (SMAW) are the best techniques for joining AISI 304, depending on the application and thickness [8]. But, the main issue with welding 304 ASS is that the high temperatures employed cause chromium carbide to develop at the grain boundaries of the heat-affected zone (HAZ), which depletes the grains of chromium and reduces their corrosion resistance.

Austenitic stainless steel sensitization is the name given to this phenomenon [14]. On the other hand, the rapid cooling rate in the RSW process reduces the amount of carbide that forms. The impact of the welding current on the microstructure and nugget diameter of the ASS 304 resistance spot-welded material was studied by Bhat et al. [15]. They deduced that, up to a certain degree, the nugget diameter rose in direct proportion to the welding current, after which it declined. Higher currents caused the HAZ grains and size to rise, and the microstructure of both the base metal and the HAZ is mostly made of austenite. But, austenite and delta ferrite were discovered to be the microstructure of the nugget zone.

Comparing the failure mechanisms of similar and dissimilar AISI 304 and AISI 430 joints, Zhang et al. [16] found some interesting findings. Their research demonstrated that when the strain concentration in the nugget was beyond its local plastic limit, the interfacial mode of the 304 similar joints was seen. In addition, by raising the equivalent stress over the critical limit, the 430 similar joints exposed the interfacial mode. Due to their stronger nugget zone, the dissimilar joints of 304 and 430 exhibited the pullout failure mode, in contrast to the similar joints discussed before. Due to the rapid cooling that occurs during small-scale resistance spot welding (SSRSW), the austenite phase becomes the dominating microstructure in the weld zone, as seen in a study on 304 ASS by Fukumoto et al. [17].

The fast solidification also suggests that the Scheffler charts cannot foretell the microstructure. It was investigated by Sun et al. [18] how the sealant affected the microstructure and failure mechanism of 301L ASS. They determined that compared to the RSW method without the use of a sealant, the addition of a sealant causes nugget initiation to occur sooner, nugget size to increase, and the heat ratio to rise. Although the mechanical properties can be improved by adjusting the increment in the welding current, the failure mode and mechanical properties were both unacceptable when a sealant was used. The nugget and heat-affected zones of 304 ASS were found to be larger as the welding current increased, according to Özyürek [19].

The morphology of delta ferrite changed from acicular to lath and skeleton; however, the weld zone comprised austenite and ferrite, according to Kianersi et al. [20], who found that this shift was caused by the cooling rate used during the RSW process. The hardness and tensile load of the 316L resistance spot-welded joints were examined by Kocabekir et al. [21] in relation to the welding time and various welding atmospheres. Joints welded in a nitrogen environment had a larger tensile load compared to those welded in a normal atmosphere, and they discovered that both the shear force and nugget size increased as the welding duration rose. When it comes to hardness, the normal atmosphere achieves its peak at a weld time cycle of 15 cycles, while the nitrogen atmosphere reaches its peak at a weld time of 20 cycles.

A study conducted by Alizadeh-Sh et al. [22] examined the relationship between thermal cycles and the microstructure of AISI 430. The results showed that many factors, such as grain growth and the formation of martensite and carbide, affect the microstructure of the heat-affected zone (HAZ) and fusion zone. In their study, Lakshminarayanan and Balasubramanian [23] demonstrated that laser beam welding of AISI 409M resulted in the formation of dendritic and equiaxed grains in the weld metal. This led to an increase in both tensile strength and impact toughness of the weld metal, surpassing that of the base metal. The FZ microstructure is completely austenitic, according to the research on the resistance spot joints of AISI 304 conducted by Marashi et al. [24]. Regardless, the microstructure of AISI 304 weld nuggets is predicted by the Schaeffler diagram to include two phases, austenite and ferrite, in the nugget zone.

Pouranvari et al. [25] examined the transition of failure modes in DP980, DQSK, DP780, DP600, and AISI 304 steel. It was noted that the probability of failure in the interfacial mode increased in the following order: DP600, DP780, DP980, DQSK, and AISI 304 steel. Midhun et al. [26] applied the Taguchi method to analyze the impact of welding current, welding time, and pressure on the dissimilar RSW joints of AISI 304 and AISI 202. The impact of varying welding currents and durations on the microstructure and mechanical characteristics of AISI 304 and AISI 430 was investigated by Khuenkaew et al. [27]. The quality of the welds was said to have improved when the welding time and current were increased. In contrast to the almost identical fusion zone (FZ) hardness of the two stainless steel types, the AISI 425 HAZ experienced a drop in hardness. Austenite, ferrite, and martensite were seen as columnar grains in the FZ microstructure.

Martensite transformation was observed during interfacial fractures in austenite stainless 301, as determined by Liu et al. [28], who examined its microstructure and mechanical performance. In line with the findings of Moshayedi and Sattari-Far [29], there exists a positive correlation between the diameter of the nugget and both the welding time and current. Among these variables, the welding current exhibits the greatest influence. In addition, residual stresses were greater in the interior nugget region compared to the peripheral one. While the nugget height of 2205 duplex stainless steel was greater than that of AISI 316L, Krishnan et al. [30] discovered that the diameter of the nuggets fell within the acceptable range. Mezher et al. [31] examined the RSW of AISI 316L using FEM and practical analysis.

Heterogeneous hardness in the fusion zone is the result of the transition from equiaxed to columnar grain morphology. A further observation was that the HAZ of duplex stainless steel was higher than that of austenitic stainless steel. Using neural network observations, Arunchai et al. [32] estimated the weld quality in the RSW process using an artificial neural network. The results demonstrated a 95% prediction accuracy for the model. Tensile shear testing, rather than peel testing, is superior for martensitic sheet steel M190, as stated by Pal et al. [33], and hardness measurements cannot be used to predict the interfacial mode. Further, the transition from plug-and-hole facture mode to interfacial mode occurred when the applied load was more than half of the yield load and vice versa when the percentage load was less than half of the yield load.

Mansor et al. [34] evaluated the dissimilar micro-resistance spot welding of austenitic stainless steel 316L and titanium alloy Ti-6Al-4V. The tensile shear test concluded that the greatest shear force for all welded specimens was 378.25 N and that they all failed in an interfacial failure (IF) mode. Mezher et al. [35] analyzed the RSW of the grade 2 titanium alloy using experimental and ANN investigations. Utilizing an ANN, Martin et al. [36] forecasted how AISI 304’s RSW parameters would affect the material’s pitting corrosion behavior. In response to their outcomes, achieving a good prediction requires careful selection of hidden layer neurons, as well as controlling overfitting during training. When comparing backpropagation with probabilistic neural network models for failure load estimation, Wan et al. [37] uncovered that backpropagation performed better. Ghafarallahi et al. [38] classified the spot weld diameter into categories using the outcomes of the finite element method (FEM) and a multi-layer perceptron (MLP) neural network model. When compared to the FEM model, the ANN model produced much more precise predictions for the weld diameter.

By varying the heat input and cooling time, Zhang et al. [39] measured the microstructure and corrosion behavior of a 304 laser-welded joint. Increased cooling time enhances corrosion resistance, and heat input significantly affects HAZ recrystallization, according to the results. Xie et al. [40] observed that the ring laser mode significantly increases the tensile strength of the ASS 304 joint up to about 96% of the base metal. Further analysis of the fractured surfaces showed the largest and densest oxide particles, which resulted in a joint elongation of 77.3% relative to the apparent metal. The microstructure of the AISI 304 RSW joint’s BM, HAZ, and NZ all responded differently to the sensation effects that occurred after welding, according to Tiedra et al. [41]. Notably, the NZ showed a greater influence rate.

The tensile strength of the ASS 304 junction was shown to increase with a larger welding current, according to Arsyad et al. [42]. Moreover, the electrode was more exposed to the welded samples due to their smoothness. Nugget diameter predictions for carbon steel and AISI 304 joints were made by Sadeghian et al. [43] using FEM and experimental investigation. Increases in both welding time and current were shown to enhance nugget diameter. When comparing seam-welded stainless steel joints to carbon steel joints, Zheng et al. [44] found that the former had a lower fracture toughness and the latter had a lower damage degradation parameter when subjected to cyclic stress.

The authors felt inspired to study and estimate the shear force and nugget diameter of resistance spot-welded joints due to the lack of clear criteria for the RSW process and the absence of a mathematical model in the literature review. Additionally, in previous publications, the effects of RSW parameters, such as welding current, pressure, welding time, squeeze time, holding time, and pulse welding on shear force, nugget diameter, micro-hardness, and failure mechanism for 1–1 mm, 0.5–0.5 mm, and 0.5–1 mm AISI 304 cases, were not conducted simultaneously. In addition, as an innovative contribution to the RSW field, a mathematical equation of tensile shear force and nugget diameter was derived using the weight and biases of the best ANN model.

## 2. Materials and Experimental Procedures

### 2.1. Materials and Welding Process

**Figure 1:**
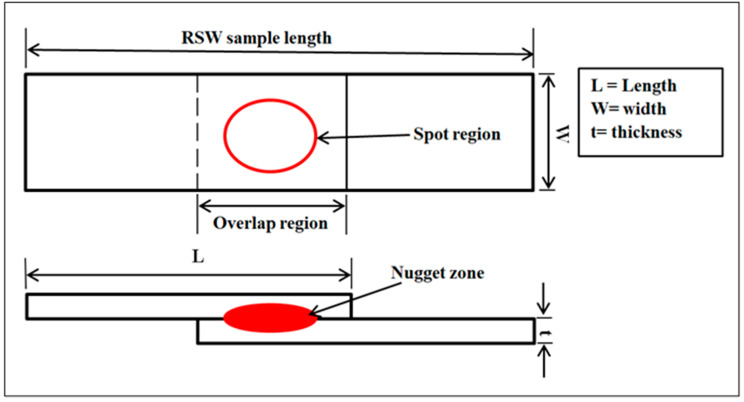
Schematic illustration of the RSW specimen.

In this study, 1 and 0.5 mm thick austenitic stainless steel AISI 304 were employed in the resistance spot welding (RSW) process. The specimens’ dimensions were taken as a lap-joint assembly, as shown in Figure 1, according to the AWS specifications [45] depicted in Table 1. Its mechanical properties and chemical composition are listed in Table 2 and Table 3, respectively. Prior to executing the RSW process, the austenitic stainless steel samples were mechanically polished using grit-abrasive paper No. 800. This was performed to eliminate any cutting-edge burr, surface contaminants, and oxide coatings that could have emerged. Weld quality determination relies on preventing expulsion formation during RSW; therefore, ethanol was employed to remove contaminants from sample surfaces that may have caused expulsion.

**Figure 2:**
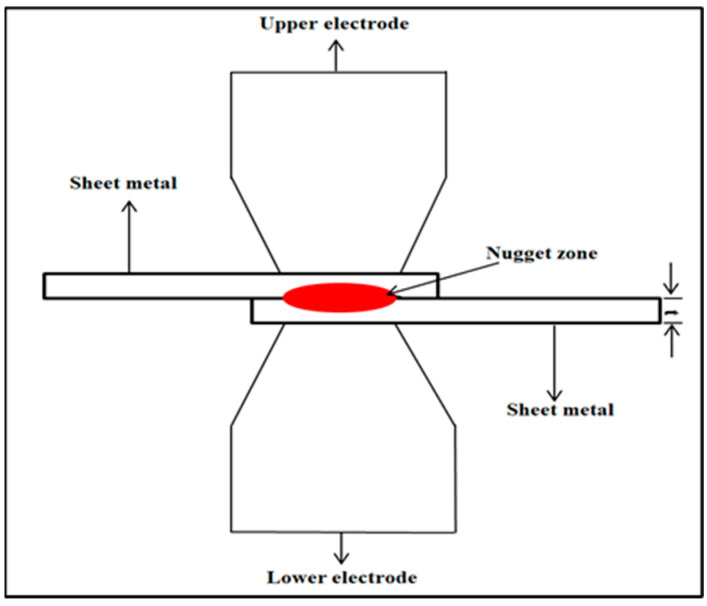
Schematic illustration of the RSW process.

**Figure 3:**
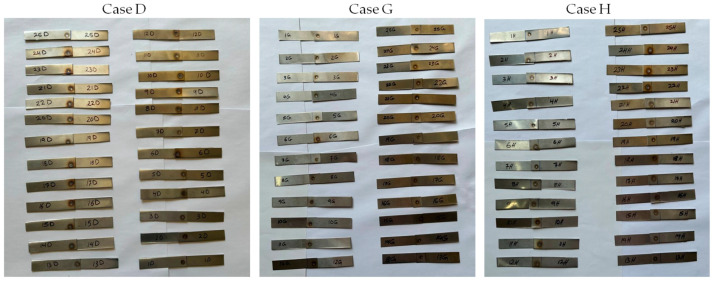
Resistance spot-welded specimens.

The RSW machine used a set of RWMA group A class 2 type B electrodes that were made of a chromium–zirconium–copper alloy. These electrodes had a spherical tip with a diameter of 4 mm. Figure 2 shows a schematic of the RSW process. The machine pressure was controlled using a pneumatic method. The RSW conditions were inputted into the machine via the digital screen. The RSW trials were conducted at ambient temperatures. The current research used the following RSW parameters: weld current of 5000–7000 A, weld time of 0.6–1.4 sec, squeeze time of 0.6–1.4 sec, hold time of 0.5–1.5 sec, pulse welding of 1–5, and pressure of 2–8 bar. An orthogonal array according to the Taguchi method using the design of the experiment (DOE) was selected to define the RSW conditions of each experiment, as revealed in Table 4. The resistance spot-welded samples of the three cases are shown in Figure 3.

### 2.2. Testing and Characterization

**Figure 4:**
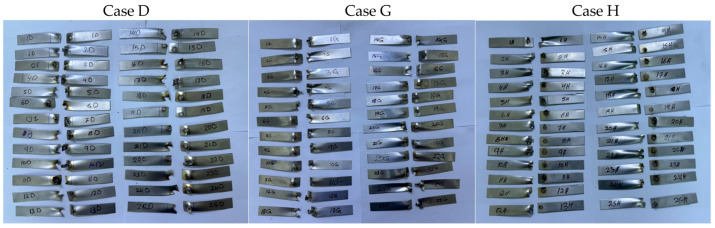
Fractured resistance spot-welded specimens after the tensile test.

Tensile shear force measurements were used to evaluate the RSW joints’ mechanical performance. The peak load required to break the RSW samples was determined by subjecting the RSW joints of 1–1 mm, case D; 0.5–0.5 mm, case G; and 1–0.5 mm, case H, to tensile shear force. For the tensile shear tests, it was a universal testing machine that could withstand a peak load of 100 kN and a cross-head speed of 5 mm/min at ambient temperature. Extracting the force–displacement curve from the tensile shear test allowed us to determine the peak load after specimen breakage. In order to evaluate the final diameter according to the ANSI/AWS/SAE standards, the nugget diameter was also measured [46]. To a large extent, the resistance spot weld quality is dictated by the nugget diameter. In order to determine whether the RSW joints failed around the fusion zone or the HAZ, the failure analysis of the tensile shear specimens was used. The samples that were fractured after the tensile shear test are shown in Figure 4.

**Figure 5:**
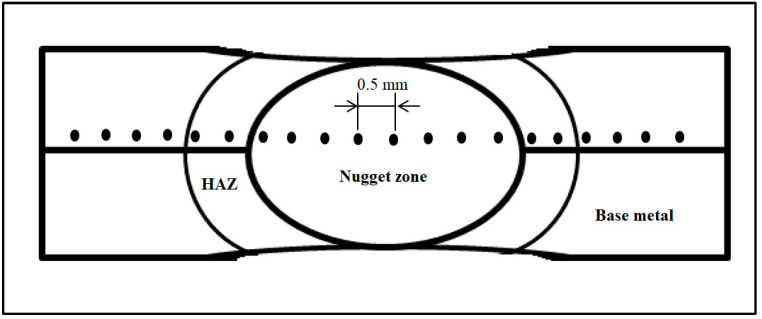
Micro-hardness positions along the weld profile.

Based on the outcomes of the tensile shear test samples, which showed the greatest, medium, and lowest shear forces, the three specimens in each instance were subjected to the micro-hardness of the RSW joints. Using a pyramidal diamond indenter tester (HVS-1000 series, Laryee Testing Machines) and a load of 2.98 N, the micro-hardness was measured using a Vickers test. The dwell period was set at 10 s. After being cut in a direction that was perpendicular to the weld surface plane through the middle of the weld nugget, all three RSW specimens were mounted in acrylic, which is a combination of resin and hardener. In addition, an etching solution consisting of 2HCl, HNO_3_, and 3H_2_O was used after conducting the polishing process for the mounted RSW joints. As illustrated in Figure 5, the micro-hardness tests were conducted along the welds at equal distances of 0.5 mm, beginning with the base metal and progressing to the HAZ, the nugget zone, and ultimately, the base metal.

## 3. Artificial Neural Network (ANN) Modeling

Instead of building their model on a set of assumptions, neural networks use data to learn and improve over time. Most people think of neural networks as oversimplified representations of how the human brain processes neural information [47,48]. Utilizing input and output data to build a model and train it to properly anticipate process dynamics is one of the many benefits of the neural network technique [49,50]. This method thrives in situations where a full comprehension of the physical mechanics is very difficult, if not impossible, to obtain, such as the welding processes. Employing training data, the neural network learns new things. The process begins with the input variables and ends with comparing the predicted output to the actual outcome. Afterwards, the network modifies the weights of the connections between the layers. When the network shows satisfactory results on the training set, the procedure is stopped. As a further step towards improved network convergence, the network may be evaluated using validation data, which do not include the training set.

### 3.1. Backpropagation (BP) Neural Network

**Figure 6:**
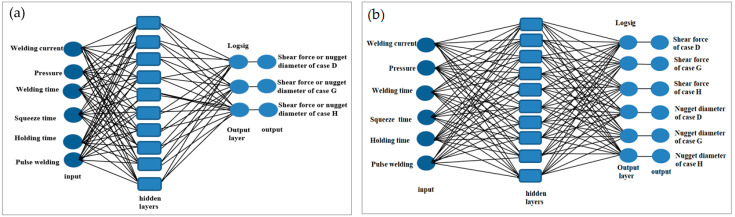
Neural network structure of the RSW process: (**a**) one output, (**b**) two outputs.

Predicting the RSW process’s output outcomes using a mathematical equation is difficult. Conversely, the RSW process may be best modeled using an artificial neural network. Neural network models may be built using a wide variety of architectures and learning techniques. Figure 6 is a flow diagram describing the three-layer backpropagation network architecture, which is the most powerful and successful network design [51]. A neural network is a computational model consisting of interconnected processing components, each with its own set of connection weights. The information is conveyed by connection weights, which are modified throughout the learning period.

Multiple algorithms may be used in neural networks, and each method can be assessed based on several validation measures, such as mean squared error and correlation coefficient (R^2^). This backpropagation (BP) network has an input layer, one or more hidden layers, and an output layer, making it a multi-layer network design. Neurons are several processing units that make up a layer. There is a connection between them via weights that are yet to be decided. The input layer is responsible for receiving data from outside sources and transmitting them to the rest of the network for processing. The input layer feeds data into the hidden layer, which processes them. The output layer takes in data processed by the network and relays them to a receiver outside the network. The optimization of the ANN is carried out using MATLAB R2021a.

Two distinct neural network models were constructed using the Neural Network Toolbox^TM^ in MATLAB to forecast the actual data acquired from the RSW joints. Both structures had the same inputs, which included weld current, weld time, squeeze time, hold time, pressure, and pulse welding. In the scope of this study, different training and transfer functions were trained in order to find the optimal neural network model. The main difference between the structures was the number of outputs. The first structure is composed of one output; in this case, either the shear force or nugget diameter as shown in Figure 6a. On the other hand, the shear force and nugget diameter are the outputs of the second structure, as shown in the pictorial representation in Figure 6b. The training parameters used to model NNs were the the learning rate (0.01), the goal (0.001), and the epoch number (1000). The following formulas provide a mathematical expression for neural network processing.

(1) $$y=f\left(net\right)=\left(\sum_{i=1}^{n}w_{i}x+b\right)$$

where y is the outputs;

f is the activation function;

*x* is the input;

*w*_i_ is the weight;

*b* is the bias.

### 3.2. Transfer and Training Functions

A neuron’s normally nonlinear transfer function processes the input to the network and produces the neuron’s output. The multi-layer perceptron (MLP) uses a large spectrum of transfer functions. Therefore, selecting the optimal transfer function relies on various conditions, including the neural network (NN) structure. Additionally, in the neural network, the totals of each layer are multiplied by weights, and the resulting weighted sums are sent via a transfer function. Transfer functions determine the output of a layer by summing the weights that were inputted into the layer. In the scope of the current work, different transfer functions were used to find the ideal NN structure. Three types of transfer functions were executed to mode the NN, including the pure linear function (*purelin*), the log sigmoid transfer function (*logsig*), and the hyperbolic tangent sigmoid function (*tansig*). The tansig, logsig, and purelin transfer functions can calculated based on Equations (2)–(4). Ultimately, the hyperbolic tangent sigmoid (*tansig*) was chosen for the output layer.

(2) $$tansig\left(n\right)=\frac{2}{1+e^{-2n}}-1$$

(3) $$logsig\left(n\right)=\frac{1}{1+e^{-n}}$$

(4) $$purelin\left(n\right)=n$$

Optimization may be used to find the best design by changing the neural network’s training function. Therefore, it is not an easy job to choose the optimum training function that will operate quickly and accurately within the context of a prediction. Thus, in order to map the output parameters, this study used several training algorithms. Training functions employed for that aim were Levenberg–Marquardt (Trainlm), scaled conjugate gradient (Trainscg), conjugate gradient with Fletcher–Reeves updates (Traincgf), conjugate gradient with Powell–Beale restarts (Traincgb), and conjugate gradient with Polak–Ribiere updates (Traincgp), BFGS Quasi-Newton (Trainbfg), resilient backpropagation (Trainrp), Bayesian regularization backpropagation (Trainbr), one-step secant (Trainoss), gradient descent backpropagation (Traingd), gradient descent with momentum (Traingdm), gradient descent with adaptive learning rate (Traingda), and gradient descent momentum and an adaptive learning rate (Traingdx).

### 3.3. Data Distribution and Validation Metrics

The results of the RSW joints can be used as targets in order to forecast the predicted outcome of wedded joints. Actual data must be divided into different subsets, including training, validation, and testing datasets. Dividing a dataset into training and testing sets may have a substantial impact on any model’s performance. According to Shahin [52], the ratio of data from distinct subsets does not have a clear connection. According to Zhang et al. [53], the dividing ratio is a major issue with datasets, and there is currently no solution for this problem in a broad scenario. Regarding the data distribution in the current work, 70% was used for training, 15% for testing, and 15% for validation, based on [54]. The actual data for the RSW joints were 75 samples extracted from the experimental work, and these data were employed as training, testing, and validation data. Training is deemed complete when the validation error starts to increase and the MSE is recorded in order to prevent the network from overlearning. The mean square error (MSE) and coefficient of correlation (R^2^) are two metrics used to measure a neural network’s effectiveness [55]. High correlation occurs when, across all datasets, the predicted and target values are very close to the line. When deciding on an optimal ANN design, lower MSE and better R^2^ values are priorities. The R^2^ value serves as a valuable measure of the correlation’s validity when comparing predicted and random results. If the value of R^2^ approaches 1, the relationship is strong, and if the value of R^2^ approaches 0, the correlation is mostly random. The mean squared error (MSE) was computed using Equation (5).

(5) $$MSE=\frac{1}{N}\sum_{i=1}^{N}(y_{p}-y_{t}{)}^{2}$$

where

*y*_p_ is the predicted value;

*y*_t_ is the target value;

*N* is the total number of experiments.

## 4. Results and Discussions

### 4.1. Weld Mechanical Performance

**Figure 7:**
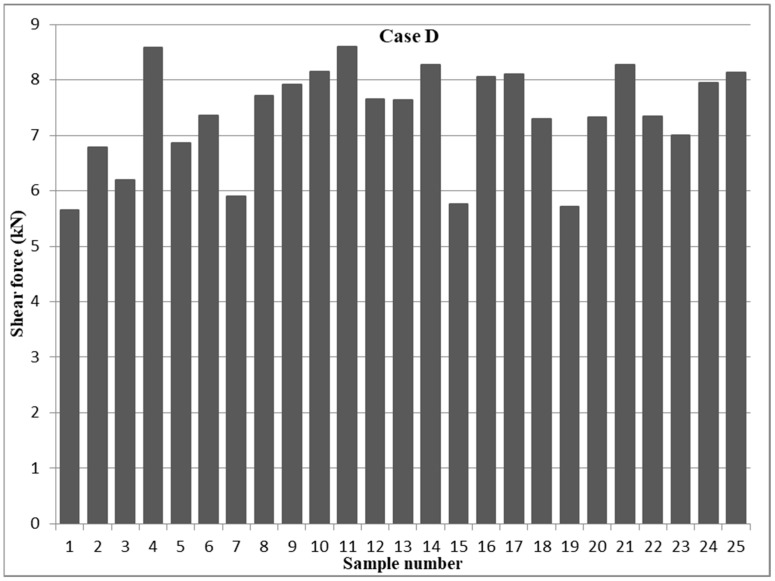
Tensile shear force results of 1–1 mm 304 austenitic stainless steel (case D).

The design of any structure that uses spot-welded joints ensures that the welds will be subjected to shear stress whenever the components are subjected to tension or compression. The welds could be exposed to shear stress, tension loading, or a combination of them, with the former direction of loading being perpendicular to the joint’s plane [56]. It is worth noting that spot welds in automotive construction might undergo shear loading from the relative movement or rotation of the neighboring sheets and tensile loading from the separation forces acting in a direction perpendicular to the sheets while performing their services [57,58]. Nevertheless, the shear force and weld nugget diameter of the RSW joints are the most crucial elements influencing the quality of the resistance spot weld [59]. Therefore, in this study, a series of 25 samples for 1–1 mm, 0.5–1 mm, and 0.5–0.5 mm cases of 304 austenitic stainless steel, as shown in Table 4, were exposed to a tensile test in order to identify the shear force of each sample. The effect of the RSW parameters on the tensile shear force results was evaluated with the aid of the design of the experiment (DOE). The DOE evaluation found that sample 11, with the following RSW parameters: a weld current of 6000 A, a pressure of 2 bar, a weld time of 1 s, a squeeze time of 1.4 s, a hold time of 0.75 s, and a pulse weld of 4, produced the maximum tensile shear force of 8.6 kN of 1–1 mm (case D). By contrast, specimen 1 demonstrated the lowest tensile shear force at 5.656 kN when subjected to the following welding parameters: a 5000 A weld current, 2 bar pressure, a 0.6 s weld time, a 0.6 s squeeze time, a 0.5 s hold time, and 1 pulse of pulse welding. Figure 7 shows the tensile shear results in case D specimens.

**Figure 8:**
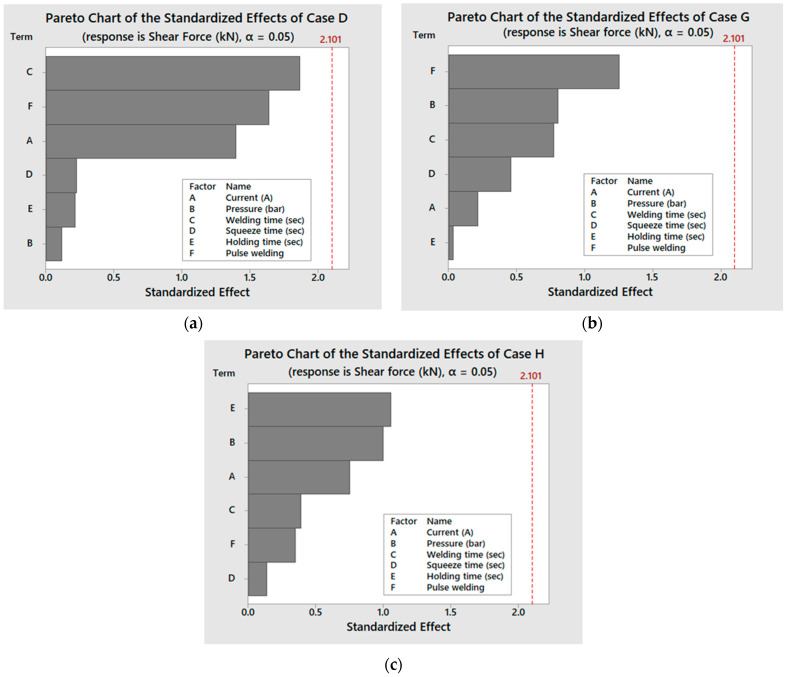
Pareto charts of the different cases: (**a**) case D, (**b**) case G, and (**c**) case H.

**Figure 9:**
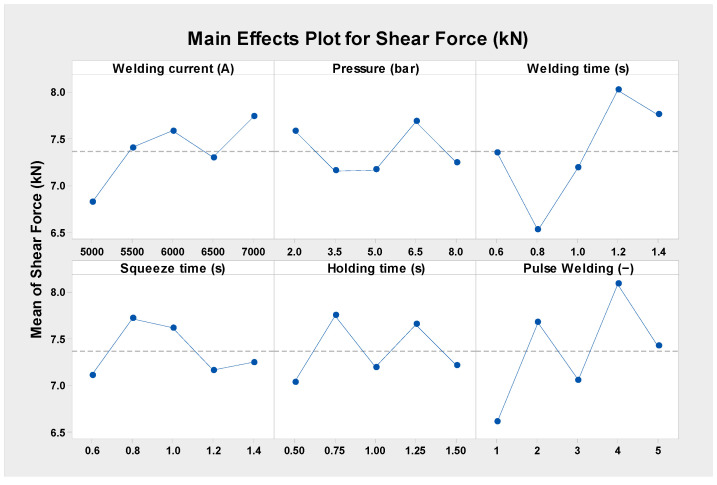
Shear force main effect plots of the RSW parameters in case D.

The weld time has the most effect on the tensile shear force in case D, with pulse welding having the second-highest influence, according to the DOE assessment of the rank response of the RSW parameters using the Pareto chart, as shown in Figure 8. In terms of their impact on the shear force, the weld current, squeeze time, and hold time are in sequence from third to fifth. Additionally, the welding pressure was shown to be the least significant contributing factor. Being the most important factor, the weld time increases the heat input and the nugget size diameter, which, in turn, increases the tensile shear force since heat transfer is a function of time according to Equation (6). Figure 9 shows the mean effect plots of the RSW.

*H* = *R* · *I*^2^ · *t* (6)

where

*H* is the heat input (J);

*R* is the resistance (Ohm);

*I* is the current (A);

*t* is time (s).

It can be shown in the mean effect plots in Figure 9 that when the welding current is increased from 5000 A to 6000 A, the shear force rises from 6.817 to 7.585 kN. However, when the welding current is increased to 6500 A, the shear force drops to 7.298 kN. However, the shear force improves to 7.739 kN when the weld current is increased to 7000. This is because when the welding current is increased, the welded samples obtain more heat input, which results in the generation of an adequate quantity of solid molten metal between the faying surfaces. The pressure welding impact main effect plots reveal that at 6.5 bar, the shear force reached its highest of 7.685 kN, while at 3.5 bar, it was recorded as the lowest, at 7.157 kN. Since the electrode pressure determines the creation of fusion zone voids, increasing the welding pressure increases the shear force. The absence of cavities or voids in the cooled weld nugget is an indication that the pressure was sufficiently high. Once the RSW joints are completed, the molten metal will solidify, which will cause pores and cavities to develop.

Due to solidification shrinkage and thermal contraction, weld metal tends to compress as it solidifies. Furthermore, because the base metal is neither heated nor melted, it underwent some contraction, but not nearly as much as the molten metal. As a result, the base metal may prevent the solidifying weld metal from further contracting, which might lead to the formation of tensile stresses later on. But, further shrinkage and pore formation in the weld metal happens if the electrode pressure is too low and the electrodes are withdrawn before the weld pool has fully solidified. Typically, these holes may be seen near the core of the weld metal, which is where the solidification process occurred last [60,61,62].

Regarding the welding time influence, it was found that incrementing the welding time from 0.6 to 0.8 s decreases the shear force 7.346 to 6.522 kN, even after raising the welding time to 1.2 s, leading the shear force reaching 8.026 kN. Upon examining the impact of the squeeze time on the shear force, it was discovered that 0.6 s produced the least shear force of 7.108 kN and that 0.8 s produced the maximum shear force of 7.715 kN. With a holding time of 0.75 s, the shear force produced by the hold time reached its maximum of 7.748 kN, while a holding time of 0.5 s yielded the lowest result. With regard to the impact of pulse welding, it was observed that increasing the pulse welding from one to two pulses raised the shear force from 6.609 to 7.674 kN; however, increasing the pulse welding to three pulses reduced the shear force to 7.054 kN. Additionally, for four pulses, the greatest shear force of 8.087 kN was recorded. The improvement in the shear force with increasing pulse welding is because of the enlargement of the nugget diameter [63].

**Figure 10:**
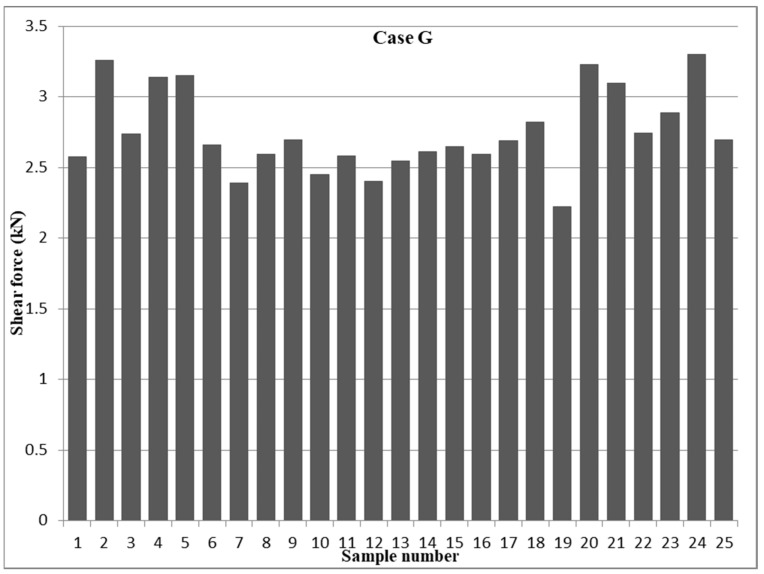
Tensile shear force results of 0.5−0.5 mm AISI 304 samples (case G).

The strongest RSW joint of 0.5−0.5 mm 304 ASS (case G), as shown in Figure 10, with a tensile shear force of 3.298 kN was confirmed by the design of the experiment (DOE) optimization employing the RSW parameters of a 7000 A welding current, a 6.5 bar welding pressure, 1 s welding time, a 0.8 s squeeze time, a 0.5 s holding time, and five pulse welding. Nevertheless, trial 7 had the lowest shear force of 2.39 kN under the following RSW conditions: 5500 A, 3.5 bar pressure, 1 s for the weld, 1.2 s for the squeeze, 1.5 s for the hold, and one pulse per weld. Examining the main effect plot in case G on the shear force with respect to the rank influence of each RSW parameter employing the Pareto chart revealed that pulse welding had the most impacting factor, as shown in Figure 8, in contrast to case D, where welding time had the most influence. Although the weld time was found to be the third factor affecting the outcome, the order of the other factors’ relative responses was as follows: pressure was second, squeeze time was third, current was fourth, and holding time was the least impacted.

**Figure 11:**
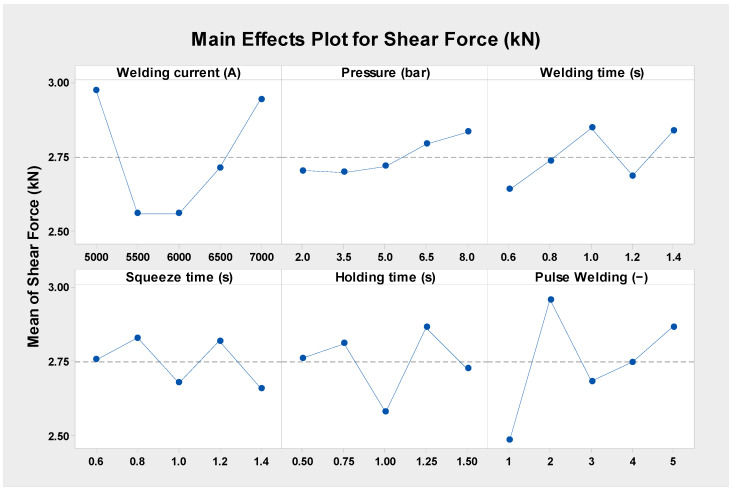
Shear force main effect plots of the RSW parameters in case G.

Figure 11 shows the main effect graphs, which indicate how the RSW components affected the shear force in case G. Importantly, the shear force peaked at 5000 A when the welding current was first measured, but it gradually decreased when the current was raised from 5500 to 6000 A, reaching its minimum value at these currents. As the welding pressure was raised, the shear force also rose, suggesting a clear link between the two variables. In terms of the results pertaining to the time of the welding process, it was first observed that there is a nearly linear relationship between the two variables, that is, as the time of the welding process increases from 0.6 to 1 s, the shear force also increases linearly until it reaches its maximum value at 1 s. However, after this peak, the shear force considerably decreases until it reaches its lowest point at 1.2 s, and then it increases linearly up to 1.4 s. In addition, the results related to the squeeze and hold times demonstrated an indirect association with the shear force. The results revealed that a squeeze time of 0.8 s and a hold time of 1.25 s had the most effect, while a squeeze time of 1.4 s and a hold time of 1 s produced the least impact. Pulse welding showed that its least impact was at one pulse per weld, while its most impact was at two pulses per weld.

**Figure 12:**
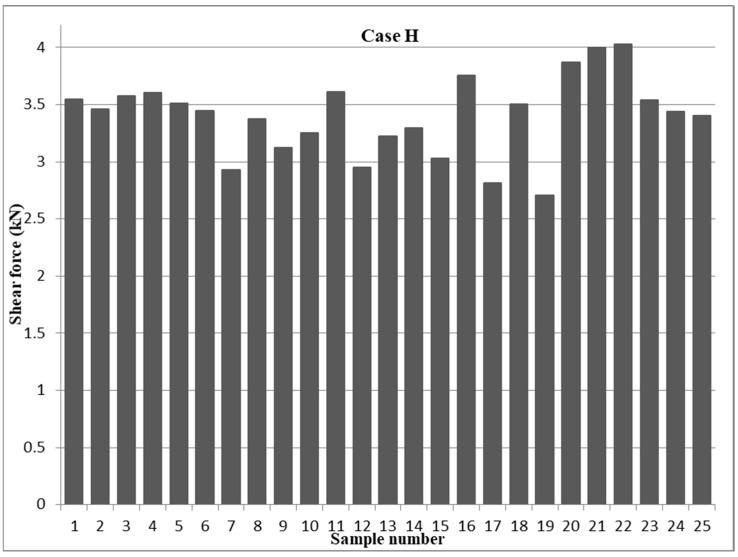
Tensile shear force results of 0.5−1 mm AISI 304 samples (case H).

With respect to the results of the tensile shear force of the 304 ASS with uneven thicknesses of 0.5 and 1 mm (case H), experiment number 22 produced a peak tensile shear force of 4.031 kN, as shown in Figure 12. The test was conducted under the following RSW conditions: 3.5 bar of pressure, 7000 A of weld current, 0.6 s of weld time, 1.4 s of squeeze time, 1.25 s of hold time, and three pulses of pulse welding. On the other hand, the DOE analysis showed that, at the RSW parameters shown in Table 4, the minimal shear force was observed with experiment number 19. By analyzing the relative impact of every welding parameter on the shear force in case H, it was found that the squeeze time had the least impact and the hold current had the most influence, as depicted in Figure 8. The remaining variables had the following relative responses: the second factor was pressure, the third was current, the fourth was welding time, and the fifth was pulse welding.

**Figure 13:**
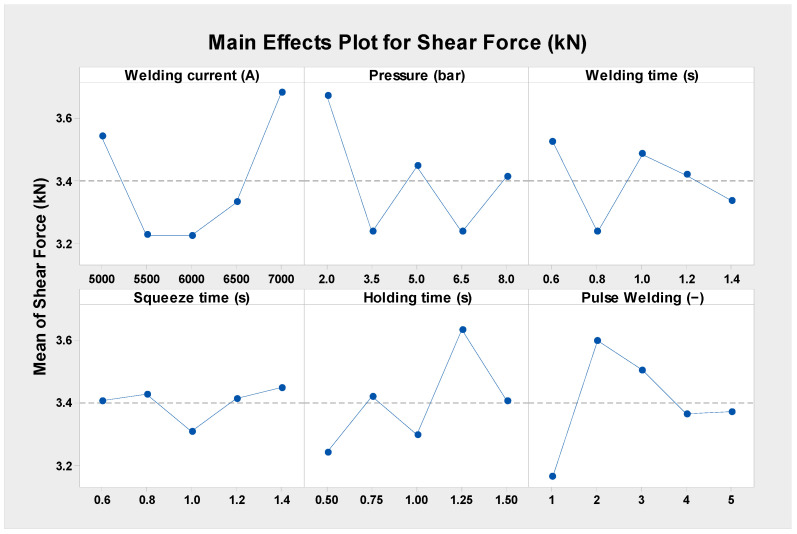
Shear force main effect plots of the RSW parameters (case H).

According to Figure 13, the weld current had the greatest impact on the shear force at 7000 A in the mean effect plots, whereas welding currents at 5500 and 6000 A had the least amount of impact. The lowest value of the mean shear force is found between pressures of 3.5 and 6.5 bar, while the maximum value is found at pressures of 2 bar. In response to the impact of welding time, the mean force was seen to be lowest at 0.8 s of welding time, while the maximal average force was computed at 0.6 s of welding time. By comparison, the maximum mean shear force is obtained with a squeeze period of 1.4 s, while the minimum effect is seen with a squeeze time of 1 s. Figure 13 shows that the most favorable mean force is obtained at a holding period of 1.25 s, while the least acceptable impact was seen at a holding time of 0.5 s. The greatest effect was seen with two pulses per weld, while the least amount of influence was produced by one pulse of welding.

### 4.2. Nugget Diameter Observations

The size of the nugget, as well as the shear force and failure modes, are often regarded as the primary indicators for evaluating the quality of a weld. The weld nugget size, also known as the diameter of the weld nugget in the longitudinal direction at the interface between the sheets, is a critical measure for assessing the quality of resistance spot welds. In general, the objective is to generate resistance spot welds with the necessary diameter that experienced pull-out failure mode during the tensile shear test. A pull-out failure (PF) mode occurs when there is a fracture in the base metal, the heat-affected zone, or the weld nugget, resulting in a metal button remaining on one of the sheets being welded. In contrast, interfacial (IF) failure refers to the occurrence of fracture specifically at the weld spot between the sheets at the plane of the interface. Typically, the necessary weld diameter is determined based on Equation (7), which is recommended by ANSI/AWS/SAE. This ensures that the weld size is sufficiently large enough to withstand the load during the shear test.

*D* ≥ 4 *t*^0.5^ (7)

where

*D* is nugget diameter (mm);

*t* is sheet thickness (mm).

The nugget diameter is the driving force behind the bonding area of the RSW joint between the sheets. The RSW parameters primarily govern the heat input, which, in turn, affects this bonding area. Thus, to find out whether the measured nugget diameter coincides with Equation (7), which is suggested by ANSI/AWS/SAE, it is essential to measure the nugget diameter of the RSW joint for the D, G, and H cases. In order to prevent interfacial failure (IF), Equation (7) states that the required diameter for a 1 mm ASS (case D) should be at least 4 mm. The nugget diameter of the majority of the RSW specimens (case D) was found to be in agreement with the ANSI formula. The D6 sample failed, partially due to both interfacial and pull-out failure modes, whereas samples D1, D15, and D19 all failed with an interfacial failure mode. The nugget diameter of 3.9 mm in sample D7 was below the acceptable value employing the ANSI formula, even though sample D7 failed with a pull-out failure type.

Table 5 shows that the optimal RSW settings for case D, with a welding current of 6000 A, a pressure of 2 bar, a welding time of 1 s, a squeeze time of 1.4 s, a holding time of 0.75 s, and a four−pulse welding, produced a nugget diameter of 5.4 mm in sample D11. The nugget diameter for 0.5 ASS (case G) should be at least 2.83 according to the ANSI formula. Applying Equation (7), the observed nugget sizes of all G samples were within an acceptable range, with the exception of the G7 sample, whose nugget diameter of 2.5 mm was below the required diameter according to the ANSI standard. Table 5 shows that the G24 sample had the biggest nugget diameter at 4.1 mm when the following RSW parameters were used: a 7000 A welding current, a 6.5 bar pressure, a 1 s welding time, a 0.8 s squeeze time, a 0.5 s holding time, and five pulse welding. It is worth mentioning that all case G samples failed under pull-out failure mode. The ANSI equation states that the accepted nugget diameter should be 3.46 mm or more when dealing with nugget diameters of 0.5−1 mm ASS (case H). Table 5 shows that while the majority of the samples had nugget diameters that were acceptable according to the ANSI formula and all of the H specimens had pull-out failure modes, specimens H6, H17, and H19 had diameters that were below what was suggested. With a welding current of 7000 A, a pressure of 5 bar, a holding time of 1.5 s, a squeeze time of 0.6 s, a welding duration of 0.8 s, and a pulse welding frequency of 4, the maximum nugget diameter was determined.

### 4.3. Artificial Neural Network Prediction

**Figure 14:**
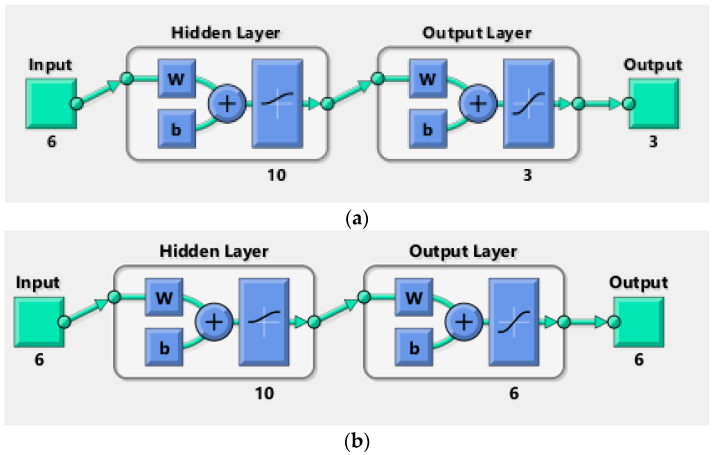
ANN structures: (**a**) one structure, (**b**) two structures.

The one- and two-output neural network structures were created using shear force and nugget diameter as the outputs to find the ideal neural network model as shown in Figure 14. The various validation metrics assessed how well each structure performed.

#### 4.3.1. One-Output Neural Network

**Figure 15:**
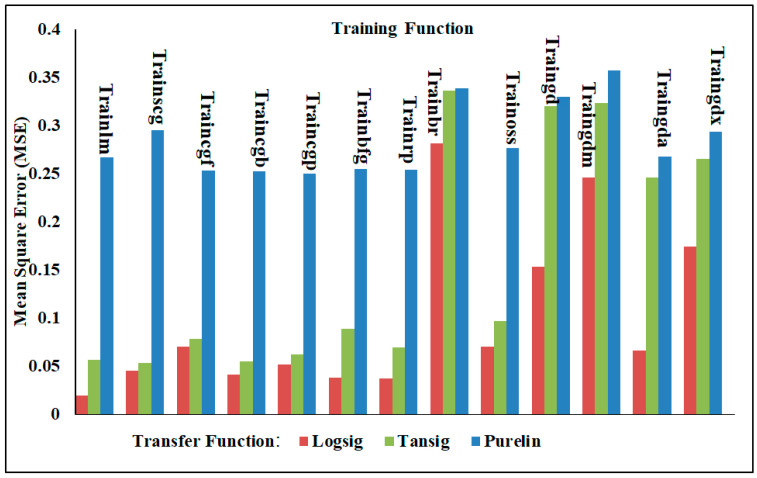
The MSE of shear force in the one-output structure using various training and transfer functions.

In this section, different neural network models were adopted to analyze the accuracy of the results for shear force and/or nugget diameter when employing the neural network with one output. Various training functions were trained with multiple transfer functions, and the findings were assessed based on the mean squared error (MSE) and correlation coefficient (R^2^) as validation metrics. Table 6 presents the two validation metrics for analyzing the shear force of the AISI 304 welded joints with the one-output neural network structure. With the MSE approaching zero and up to one in relation to R^2^, it is clear in Table 6 that the Levenberg–Marquardt (Trainlm) training function with the Logsig transfer function produced the best results. The findings shown in Table 6 and Figure 15 clearly show that the MSE and R^2^ validation metrics varied significantly within the various transfer and training functions. When trained with all training functions, the Logsig transfer function emerged the best. Conversely, the Purelin transfer function gave the lowest prediction when trained with all training functions. The MSE and R^2^ of Trainlm with Logsig were 0.01908 and 0.99788, respectively; however, when Trainlm was used with the Purelin transfer function, the values were 0.26678 and 0.97076. It is important to note that the Trainbr training function with these three transfer functions showed the worst performance of the NN models, with the MSE and R^2^ with Logsig being 0.28144 and 0.97185, respectively. These validation metrics performed poorly when using Purelin, with Trainbr achieving an MSE and R^2^ of 0.338416 and 0.96205, respectively.

**Figure 16:**
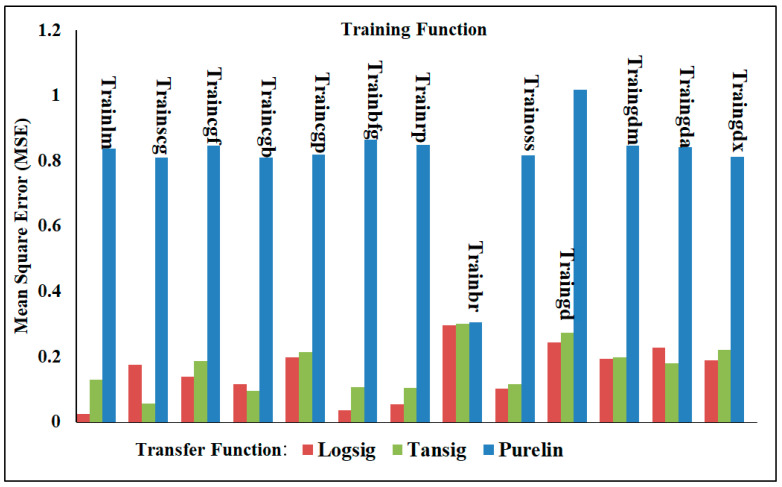
The MSE of nugget diameter in the one-output structure using various training and transfer functions.

In relation to the one-output ANN’s prediction of the nugget diameter of the welded joints, Table 7 emphasizes the difference between MSE and R^2^ for all training and transfer functions. The performance of the training and transfer functions in predicting the nugget diameter with respect to the validation metrics MSE and R^2^ was less than these validation metrics when predicting the shear force at the same training and transfer functions, as demonstrated by the results displayed in Table 7 and Figure 16. The best results were obtained using the Trainlm training function with Logsig, which achieved MSE and R^2^ values of 0.02580 and 0.99091, respectively, while using Purelin with Trainlm, the values were 0.83903 and 0.7047, respectively.

**Figure 17:**
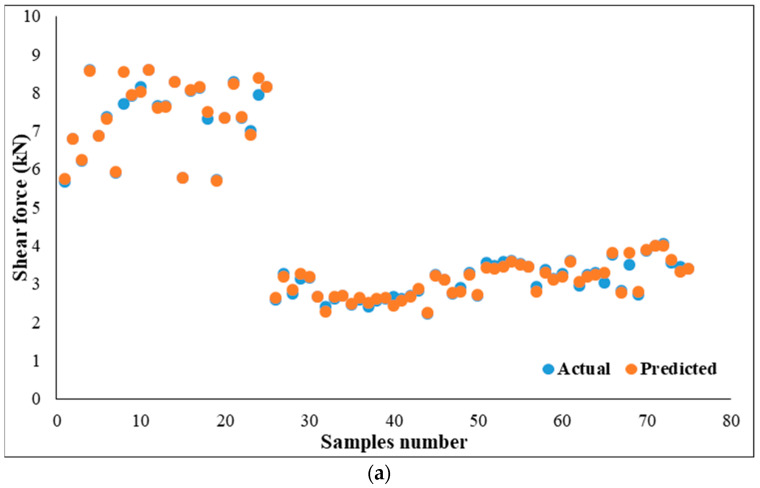

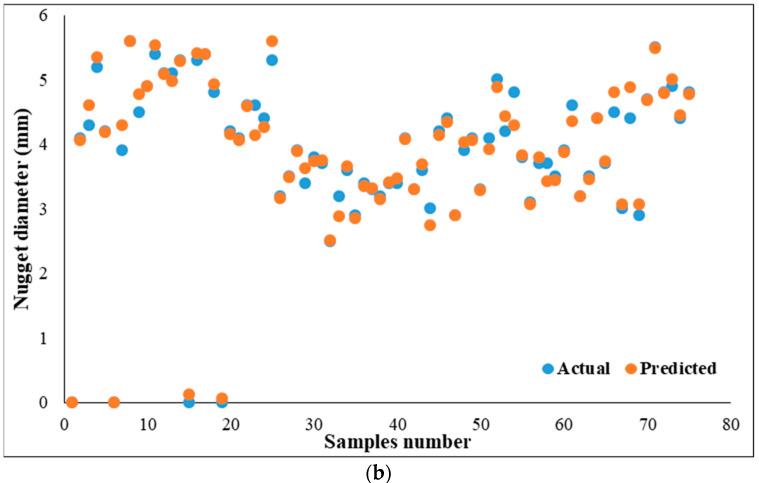
Actual and predicted results: (**a**) shear force, (**b**) nugget diameter.

Conversely, the least accurate predictions of the nugget diameter neural network models were recoded using the Purelin and the Traingd training function, and the resulting MSE and R^2^ values were 1.01768 and 0.65619, respectively. The decreased performance of the nugget diameter models compared to the shear force models could be associated with the failure of some experimental RSW samples in the interfacial mode. Consequently, when constructing neural network models, the nugget diameter was considered to be zero for these specimens. For this reason, a fluctuation in prediction occurred for each output, which is located far from each of them. This is interpreted by the fact that the nugget diameter values range from 0 to 5.6 mm, which impacts the performance of the MSE and R^2^ validation metrics for all neural network models. Moreover, Figure 17a,b demonstrated a considerable positive correlation between predicted and actual datasets of shear force and nugget diameter.

#### 4.3.2. Two-Output Neural Network Model

**Figure 18:**
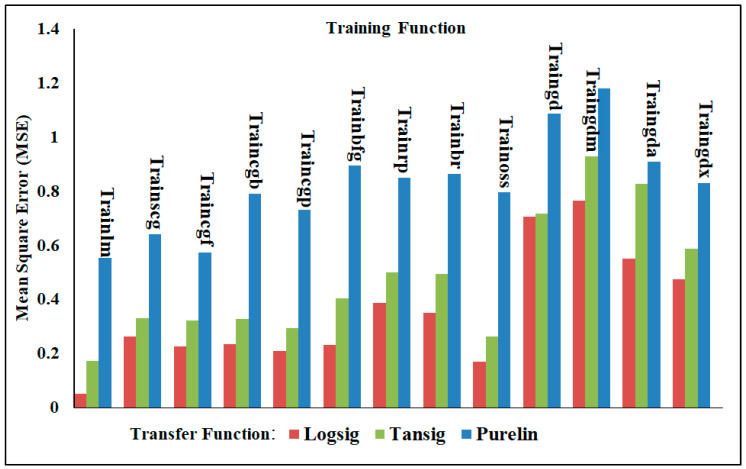
The MSE of the nugget diameter and shear force in the two-output structure using various training and transfer functions.

The performance of the neural network models was evaluated in this part using a two-output neural network structure. This means that both the shear force and nugget diameter were predicted using the same artificial neural network models and trained with the same training and transfer functions as the one-output structure. From the data shown in Table 8 and Figure 18, it is clear that Trainlm with Logsig achieved the best performance, with the MSE and R^2^ being 0.05172 and 0.99183, respectively, while they decreased to 0.55255 and 0.91556 when Trainlm was trained with Purelin. On the contrary, Traingdm gave the least performance when measuring the MSE and R^2^ with the different transfer functions. The MSE and R^2^ for Traingdm with Logsig were 0.76383 and 0.86808, respectively, whereas these validation metrics were lowered to 1.1785 for the MSE and 0.7926 for R^2^ when Purelin was used with Traingdm. An important point to keep in mind is that the prediction of the shear force in a one-output structure is more accurate than that employing a two-output structure model; however, the prediction of the nugget diameter in a one-output structure is least accurate compared to the prediction utilizing a two-output structure. Among the most noteworthy findings from the ANN results is the fact that the one-output structure’s nugget diameter prediction using the Purelin transfer function performed significantly worse in terms of the evaluation indicators, MSE and R^2^, when compared to the two-output structure’s predictions using the same transfer function.

**Figure 19:**
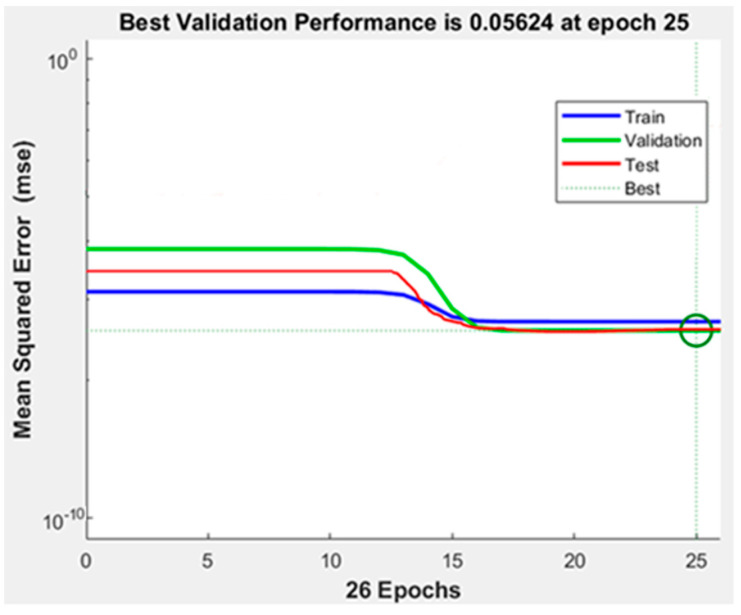
Performance curve of a one-output ANN model of shear force using Trainlm with Logsig.

**Figure 20:**
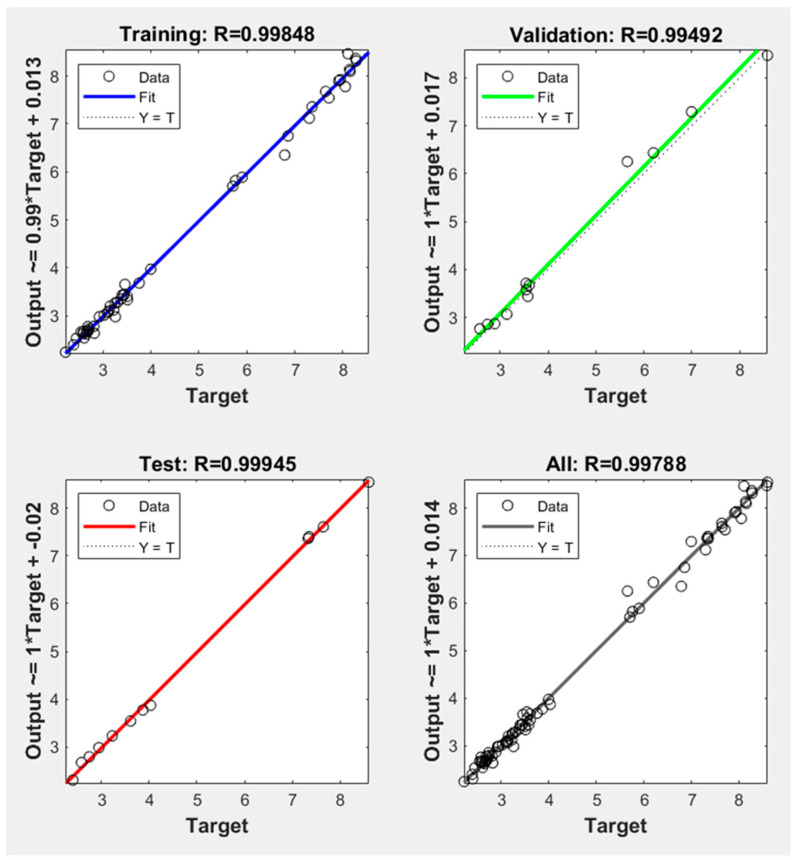
Regression curve of actual and predicted data of the one-output ANN model of shear force using Trainlm with Logsig.

In addition to the MSE and R^2^ indicators, additional validation metrics were used to evaluate the best neural network model of the one and two structures. The following metrics are used for evaluation: the mean absolute error (MAE), mean relative error (MRE), root-mean-squared error (RMSE), and mean error (ME). All the assessment indicators for the best ANN using Trainlm with Logsig are shown in Table 9. Figure 19 and Figure 20 show the best performance and actual and predicted data curves of the optimal model when using the Trainlm training function with the Logsig transfer function. Figure 19 indicates that the mean squared error (MSE) through the training, validation, and testing stages decreases with increasing the epoch until they stabilize in the same trend, meaning that the ANN model is well trained and fits the data according to the approved standards.

#### 4.3.3. Mathematical Equations

As a new contribution to the RSW field for the first time, the analytical equations for predicting the shear force and nugget diameter were derived based on the weights and biases extracted from the best ANN model of the shear force and nugget diameter. Since the shear force and nugget diameter ANN models used a single hidden layer, there was only a single set of input weights (IWs) and layer weights (LWs) in this work. A layer weight (LW) is located between the outputs and the hidden layer, while an input weight (IW) is situated between the input parameters and the hidden layer. Not only that, but b1 and b2 were the biases assigned to each layer. Table 10 and Table 11 provide the IW, LW, b1, and b2 generated from the optimal ANN model concerning the shear force and nugget diameter, respectively.

(8) $$logsig\left(n\right)=\frac{1}{1+e^{-n}}$$

(9) $$y\;f\left(net\right)=\left(\sum_{i=1}^{n}w_{i}x+b\right)$$

Since *y* represents the output and the outputs in this paper are the shear force or the nugget diameter, Equation (9) becomes as follows:

(10) $$\mathrm{Shear}\;\mathrm{force}\;\mathrm{or}\;\mathrm{nugget}\;\mathrm{diameter}=f\left(net\right)=\left(\sum_{i=1}^{n}w_{i}x+b\right)$$

Since the $f\left(net\right)=Logsig\;(n)$, the following equations can be derived:

(11) Shear force=b2+LW × Logsig (b1+IW × x)

(12) $$\mathrm{Shear}\;\mathrm{force}=b2+\mathrm{LW}\;\times \;\frac{1}{1+exp(-(b1+IW\times x))}$$

Since the inputs (*x*) represent welding current, pressure, welding time, squeeze time, holding time, and pulse welding, the equation becomes

(13) $$\mathrm{Shear}\;\mathrm{force}=b2+\mathrm{LW}\;\times \;\;\frac{1}{\begin{array}{c} 1+exp\;(-(b1+IW\times x\;(\;welding\;current\\ \;\;\;\;\;\;\;\;\;\;\;\;\;\;\;\;\;\;\;\;\;\;\;\;\;\;\;\;\;\;\;\;\;\;\;\;\;\;\;\;\;\;\;\;\;\;welding\;time\\ \;\;\;\;\;\;\;\;\;\;\;\;\;\;\;\;\;\;\;\;\;\;\;\;\;\;\;\;\;\;\;\;\;\;\;\;\;\;\;pressure\\ \;\;\;\;\;\;\;\;\;\;\;\;\;\;\;\;\;\;\;\;\;\;\;\;\;\;\;\;\;\;\;\;\;\;\;\;\;\;\;\;\;\;\;\;\;\;holding\;time\\ \;\;\;\;\;\;\;\;\;\;\;\;\;\;\;\;\;\;\;\;\;\;\;\;\;\;\;\;\;\;\;\;\;\;\;\;\;\;\;\;\;\;\;\;\;\;squeeze\;time\\ \;\;\;\;\;\;\;\;\;\;\;\;\;\;\;\;\;\;\;\;\;\;\;\;\;\;\;\;\;\;\;\;\;\;\;\;\;\;\;\;\;\;\;\;\;\;\;pulse\;welding))) \end{array}}$$

where b2, b1, IW, and LW values are shown in Table 10.

(14) $$\mathrm{Nugget}\;\mathrm{diameter}=b2+\mathrm{LW}\;\times \;\;\frac{1}{\begin{array}{c} 1+exp\;(-(b1+IW\times x\;(\;welding\;current\\ \;\;\;\;\;\;\;\;\;\;\;\;\;\;\;\;\;\;\;\;\;\;\;\;\;\;\;\;\;\;\;\;\;\;\;\;\;\;\;\;\;\;\;\;\;\;welding\;time\\ \;\;\;\;\;\;\;\;\;\;\;\;\;\;\;\;\;\;\;\;\;\;\;\;\;\;\;\;\;\;\;\;\;\;\;\;\;\;\;pressure\\ \;\;\;\;\;\;\;\;\;\;\;\;\;\;\;\;\;\;\;\;\;\;\;\;\;\;\;\;\;\;\;\;\;\;\;\;\;\;\;\;\;\;\;\;\;\;holding\;time\\ \;\;\;\;\;\;\;\;\;\;\;\;\;\;\;\;\;\;\;\;\;\;\;\;\;\;\;\;\;\;\;\;\;\;\;\;\;\;\;\;\;\;\;\;\;\;squeeze\;time\\ \;\;\;\;\;\;\;\;\;\;\;\;\;\;\;\;\;\;\;\;\;\;\;\;\;\;\;\;\;\;\;\;\;\;\;\;\;\;\;\;\;\;\;\;\;\;\;pulse\;welding))) \end{array}}$$

where b2, b1, IW, and LW values are shown in Table 11.

### 4.4. Failure Mechanism

**Figure 21:**
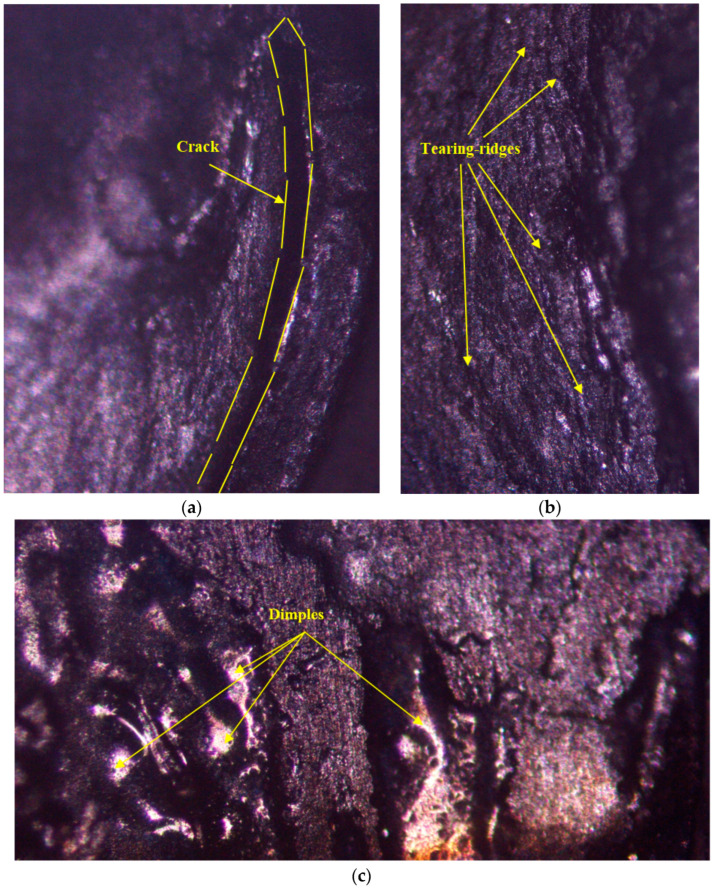
Microscope inspection of the interfacial failure mode: (**a**) crack observation, (**b**) tearing ridges, and (**c**) dimples in the fractured surface.

The mechanical characteristics of RSW joints are qualitatively impacted by their failure mechanism [25,64,65,66,67]. Spot welds are known to fail in four different ways, which are as follows [68,69]: interfacial failure (IF), wherein the fusion zone (FZ) served as the initial source of the fracture. This sort of failure is thought to have a significant effect on an automobile’s crashworthiness. The spot welds failed by withdrawing the weld nugget from the other sample, a mode known as pullout failure (PF). Under certain load conditions, the welding area’s mechanical and metallurgical qualities determine whether the fracture starts in the base metal (BM), the heat-affected zone (HAZ), or the HAZ/FZ. With regard to mechanical characteristics, the PF type is the most desirable. The partial interfacial (PIF) mode, in which the fracture expands in the thickness direction after initially propagating in the FZ. The fracture propagates in the FZ and then moves to the width, where the ultimate failure takes place in the partial pullout–partial width (PP-PW) failure mode. The current study found many failure modes that were dependent on the resistance spot welding (RSW) conditions and the thickness of the sheet, whether it was equal or not. In regard to the fracture modes of 1 mm thickness (case D), most of the samples failed with the pull-out failure (PF) mode, except for the D1, D15, and D19, which failed with the interfacial (IF) mode, as shown in Figure 21, which represents the microscope examination of the IF zone. According to the microscope inspection in Figure 21a, it is apparent that the failure is brittle and the failure is generated in the weld nugget edge, meaning that this area is more brittle than the base metal and HAZ. Further examination of the IF in the nugget area reveals that the full area consists of tearing ridges and cleavage facets, as confirmed in Figure 21b. These microscope observations demonstrate brittle fracture generation. Examining another part of the nugget area showed the presence of elongated dimples, as noted in Figure 21c, demonstrating brittle fracture occurrence.

**Figure 22:**
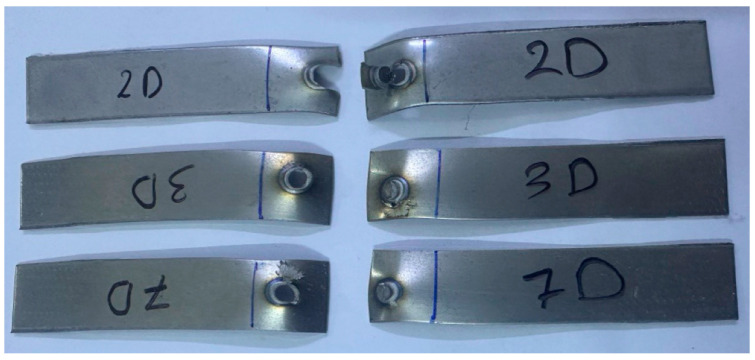
Pull-out failure mode of some samples in case D.

The mechanism of the pull-out mode was not the same in all samples, where the location of completing failure was different. D2, D3, and D7 failed, with the full pull-out nugget starting around the HAZ and then ending by withdrawing all of the nugget zones from the other side of the sheet, as confirmed in Figure 22.

**Figure 23:**
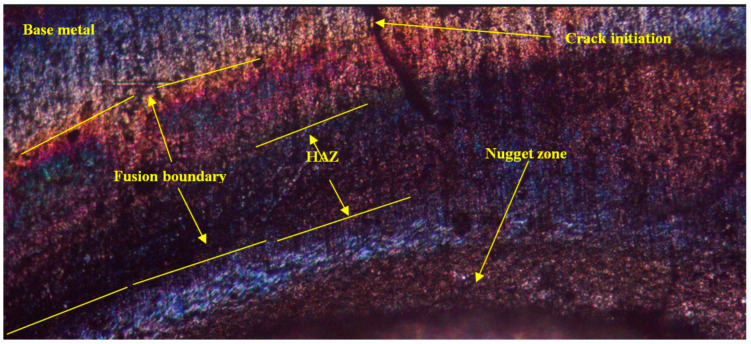
Micro-examination of the pull-out failure region.

**Figure 24:**
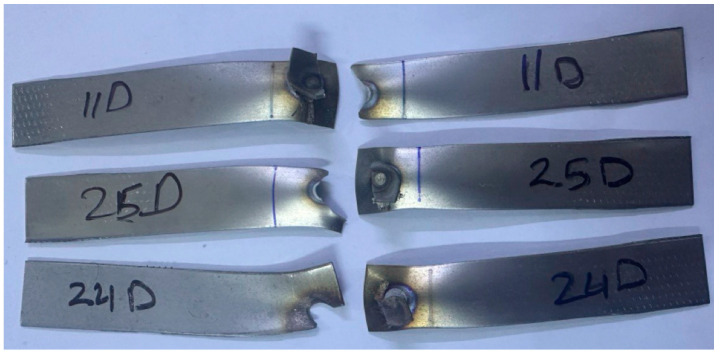
Crack transferring through the width in case D.

Micro-examination of pull-out failure showed that the crack initiated outside the nugget area, mostly in the base metal, as confirmed in Figure 23, meaning that the nugget area is stronger than the base metal and HAZ due to more depth indentation caused by more heat generated by higher current flow through the welding electrodes. On the other hand, in the other spot welds that experienced pull-out failure (PF), the fracture started around the HAZ, but later, the crack transferred to the width where the final fracture was observed, as noted in Figure 24.

**Figure 25:**
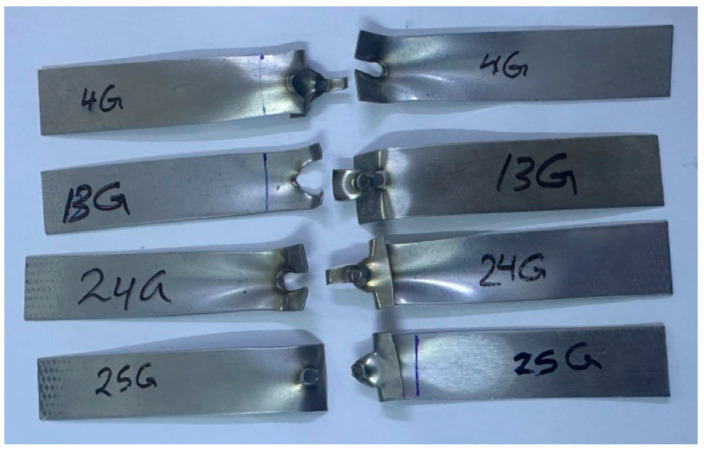
Complete withdrawal of the nugget in case G.

**Figure 26:**
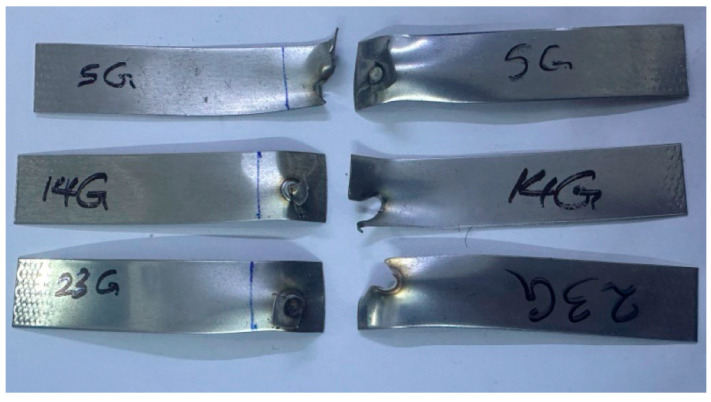
Crack transferring through the width in case G.

Regardless of whether the failure ended via the width or the nugget’s full withdrawal, the pull-out failure (PF) type was the most prevalent type of failure for the RSW joints of the 0.5 mm 304 ASS (case G) in all test samples. Figure 25 shows that for the samples (1, 8, 11, 17–22), G failed due to the complete withdrawal of the nugget zone from the other sheet. According to Figure 26, the failure mechanism of the other samples was a pull-out nugget as well. In this case, however, the failure began at the HAZ and progressed along the width until the ultimate fracture was witnessed.

**Figure 27:**
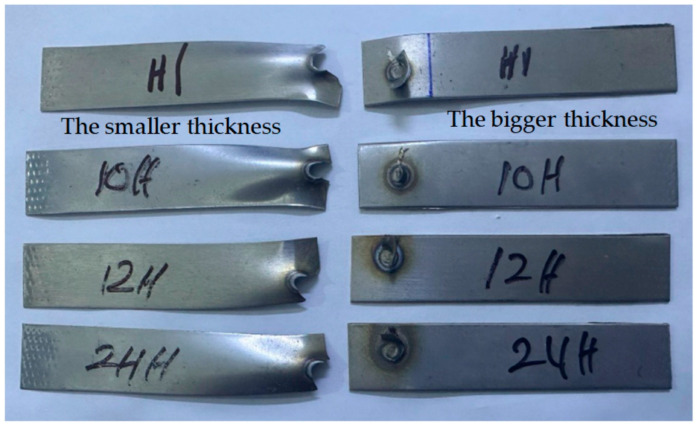
Nugget withdrawal in pull-out failure mode in case H.

With respect to case H, which involved 0.5–1 mm 304 ASS, it was observed that all RSW specimens experienced pull-out failure (PF) mode, although with variations in the manner in which the fracture was completed. Certain samples, including 7–10, 13, 14, 17, and 19 H, experienced PF type due to the full withdrawal of the nugget zone from another sheet. Nonetheless, the PF mode was also seen in the remaining samples, and the fracture, which originated near the HAZ, extended and terminated across the width. It is noteworthy to point out that in case H, the thicker sheet (1 mm) withdraws the nugget zone from the thinner sheet (0.5 mm) during the PF mode, as shown in Figure 27.

**Figure 28:**
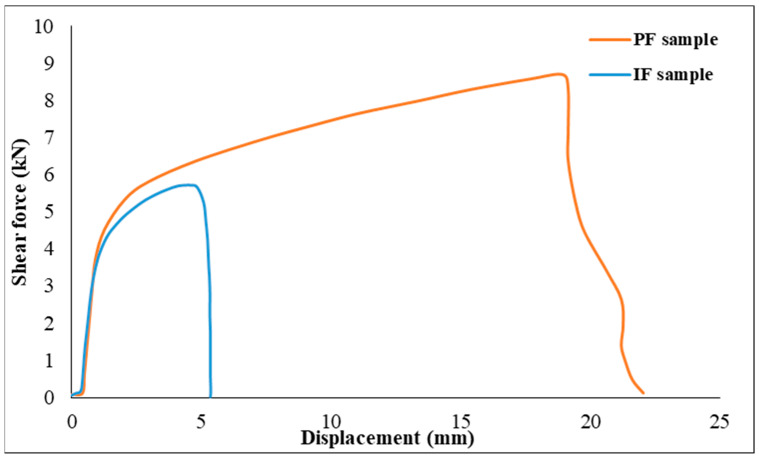
Correlation between shear force and failure mode type.

Intriguingly, the manner of failure may significantly affect the RSW joints’ load-bearing capability and energy absorption ability. With its maximum plastic deformation and energy absorption, the pull-out failure mode stands out as a very desirable failure type [70,71]. Crashworthiness is a key consideration in vehicle design; hence, in cases when RSW joints fail via the interfacial failure (IF) mode, crashworthiness may be significantly reduced. It is worth noting that the joints can withstand large loads, as shown by the PF mode during quality control, which leads to significant plastic deformation in the nearby components. Experimental results from tensile shear support this claim: samples of interfacial failure (1D) had a tensile shear force of 5.656 kN, but samples of pullout failure, particularly 11D, exceeded 8.6 kN. Figure 28 further supports the idea that the PF specimen underwent more extension than the IF sample and this agreed with the results found by [71]. It can be concluded that the fracture energy is considerably impacted by the mode in which the RSW joint fails. The crack propagation path in the PF mode dissipates more plastic deformation and energy, which is desirable for the IF type, whose crack does experience a little energy and plastic deformation.

### 4.5. Micro-Hardness Distribution

It is crucial to assess the micro-hardness distribution along the welded joints’ resistance point. Different phases will develop and be split into three primary zones: base metal, the heat-affected zone (HAZ), and the nugget zone (NZ) due to the varying heat production experienced by the welding region. As a result, the hardness values along these zones will vary. In addition, the hardness of the welded joints determines the failure mode; if the nugget zone and heat-affected zone are controlled to a certain extent by decreasing their hardness to a level that is neither lower nor higher, the weld joint would not fail in the interfacial mode. The micro-hardness Vickers test, which includes 20 indentations across the welded joint with a 0.5 mm interval between each indent, was employed to perform the hardness test with a load of 2.98 N.

In the current work, the hardness measurements were implemented on the three selected welded samples in cases D, G, and H. These three specimens were selected based on the tensile shear force observations by taking the specimens that revealed the peak, middle, and minimum shear force, respectively. The hardness profile started at the base metal on one side, transferring to the heat-affected zone (HAZ) and nugget zone (NG), advancing to the heat-affected zone (HAZ) on the other side, and finally traversing through the base metal.

**Figure 29:**
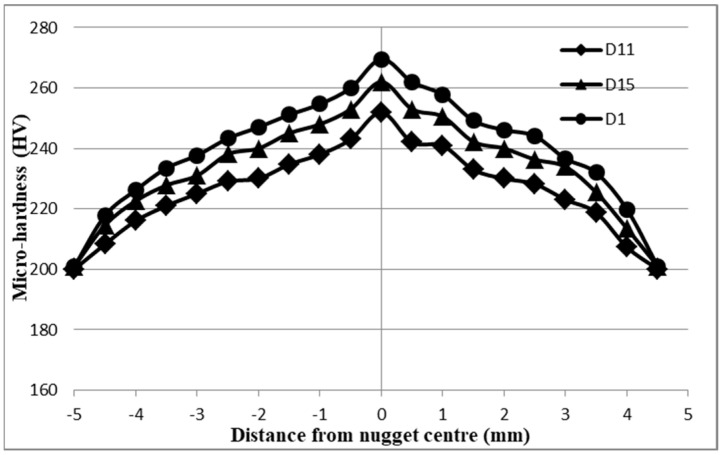
Micro-hardness results of 1−1 mm 304 ASS (case D).

Figure 29 displays the Vickers hardness measurement of the 1 mm ASS (case D). The figure illustrates a progressive rise in micro-hardness from the base metal (BM) to the heat-affected zone (HAZ), ending in the nugget zone (NG), which exhibited the maximum measured hardness. Subsequently, the micro-hardness showed a progressive decline until it reached its lowest level within the nugget zone (NG) on the opposite side. Figure 29 demonstrates that sample D1 had the highest micro-hardness among all the specimens in D. This indicates that it had the lowest shear force and experienced an interfacial failure mechanism. It is noteworthy that the interfacial mode occurred as a result of the brittle behavior of the welded joints, leading to the attainment of the highest degree of hardness. Conversely, sample D11 had the lowest hardness but demonstrated the greatest shear force and pull-out failure mode.

Upon closer examination of the micro-hardness curve of the D1 sample, it was found that the nugget zone (NZ) exhibited the highest Vickers hardness of approximately 269.5 HV. The base metal (BM) displayed the lowest hardness of about 200 HV, while the heat-affected zone (HAZ) had a hardness value of approximately 217.8 HV, which fell between the values of the BM and NZ. When it comes to the hardness distribution of sample D11, which exhibited the maximum shear force, it was observed that this sample had the lowest micro-hardness values in the NG and HAZ compared to the D1 and D15 samples. The NZ yielded a hardness value of 251.9 HV, the HAZ had an HV of 216, and the BM had an HV of 201. The Vickers hardness distribution of sample D11 exhibited a comparable pattern to that of samples D1 and D15. Nevertheless, the micro-hardness measurements of the D11 curve indicated hardness values that fell between those of the D1 and D15 curves, as seen in Figure 29. The D1 sample had a peak hardness value of 262 HV at NZ, while the HAZ had a hardness of about 222.6 HV, and the BM zone had a hardness of 200 HV.

**Figure 30:**
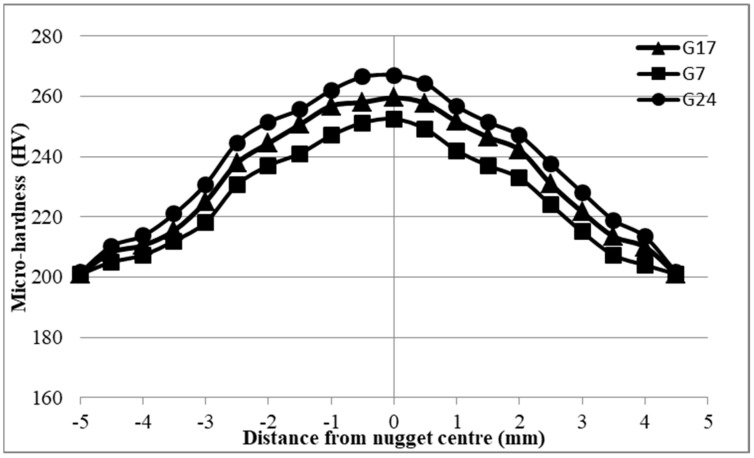
Micro-hardness results of 0.5−0.5 mm 304 ASS (case G).

Upon analysis of the hardness variations in the 0.5 mm 304 ASS (case G), it was observed that the hardness distribution in the NZ exhibited significant enhancement in comparison to the BM. Figure 30 illustrates that sample 24 had the greatest hardness values across all samples in case G, while having the lowest shear force. Conversely, sample G7, which had the highest shear force, showed the lowest hardness distribution. Furthermore, sample G17 exhibited hardness values that were intermediate between the hardness curves of G24 and G7. According to Figure 30, the hardness measurements of sample G24 ranged from 267 HV at the NZ to 201.5 HV at the BM, with a micro-hardness of 221 HV observed in the HAZ. Sample G7 had a micro-hardness of 201 HV at the BM and 212 HV at the HAZ, with the NZ recording the greatest hardness measurements of 252 HV. Earlier, it was noted that the Vickers measurement curve for sample G17 at the NZ, HAZ, and BM is situated between the curves for samples G7 and G24.

The increase in Vickers hardness may be ascribed to the hard and brittle nature of the resistance spot-welded joint. As stated by Reference [34], this suggests that there is an inverse relationship between micro-hardness and the tensile shear force of the joints. The enhanced hardness seen in the nugget zone (NZ) relative to the heat-affected zone and base metal may be due to the different cooling rates encountered by these zones. During the RSW process, the cooling rates exhibit a progressive transition from the nugget zone, located in the center of the weld where the heat source is most intense, to the heat-affected zone (HAZ), which is situated at more distance from the heat source, and finally to the base metal, which is the farthest zone from the heat source. Due to this factor, the nugget zone experiences faster rates of cooling compared to other areas, leading to an increase in the hardness of the nugget zone.

**Figure 31:**
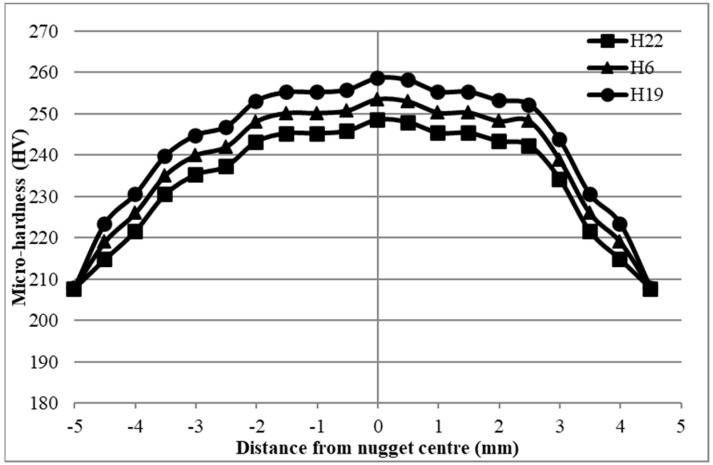
Micro-hardness results of 0.5−1 mm 304 ASS (case H).

The analysis of micro-hardness in the 0.5–1 mm unequal thickness (case H) shown in Figure 31 yielded similar findings as those seen in cases D and G. The nugget zone exhibited the maximum level of hardness, which was subsequently reduced towards the heat-affected zone (HAZ) region. Compared to the base metal, which had the lowest micro-hardness, the HAZ hardness is the most favorable. Moreover, similar to the D and G examples, sample H19 had the highest hardness while having the lowest shear force, whereas sample H22 had the lowest hardness despite having the highest shear force. One intriguing aspect of an unequal thickness curve for hardness is that the left-hand trend for thinner (0.5 mm) thickness is different from the right-hand trend for thicker (1 mm) thickness. Along the same lines, the lower thickness trend shows fewer hardness observations than the higher thickness trend.

## 5. Conclusions

In order to enhance the welding quality results of AISI 304 resistance spot-welded joints for 1–1 mm, 0.5–0.5 mm, and 0.5–1 mm cases, this paper performed experimental and artificial neural network (ANN) analysis on the effect of RSW parameters like welding current, pressure, welding time, squeeze time, holding time, and pulse welding on the quality of the resultant welded joints in terms of the tensile shear force, nugget diameter, micro-hardness, and failure mechanism. The findings concerning the shear force showed that the maximum generated shear force is considerably impacted by the thickness combination of the samples, wherein the 1–1 mm case showed the highest tensile shear force up to 8.6 kN. The case with an unequal thickness of 0.5–1 mm demonstrated a shear force somewhat greater than the case of 0.5–0.5 mm that was still lower than the case of 1–1 mm, with a recorded value of 4.031 kN. In comparison, the greatest shear force for the case of 0.5–0.5 mm was 3.298 kN. In addition, the research showed that the nugget diameter is a significant factor in determining whether the failure mechanism is pull-out or interfacial. In light of this, the ANSI formula was used to assess the three examples’ nugget diameter findings. The majority of the case D samples had nugget diameters within the acceptable range, given by the ANSI formula. However, there was one sample that went down a little from the required value; fortunately, it failed in pull-out failure mode. The same observations were noted with the cases of 0.5–0.5 mm and 0.5–1 mm. While most of the samples emerged with an accepted criterion of the nugget diameter, one sample from the 0.5–0.5 mm case and three samples from the 0.5–1 mm case were lower than the given ANSI value. The results showed that ANN models were crucial for predicting how the RSW parameters would affect the shear force and nugget diameter. The ANN models demonstrated that although it is preferable to use the one-output structure for shear force rather than two for all training and transfer functions, it was better to use two for nugget diameter prediction. Additionally, using the Trainlm training function with the Logsig transfer function results in the best performance when it comes to validation measures, like the MSE and R^2^. In fact, for the first time in the RSW field, this paper proposes two equations for predicting the shear force and nugget diameter based on the RSW parameters. Finally, the micro-hardness of the highest, medium, and lowest shear force samples was measured, and the results showed that the highest shear force sample gave the lowest hardness, while the highest hardness was noticed with the lowest shear sample for the three cases.

## Figures and Tables

**Table 1:** Dimensions of the RSW specimens.

Thickness (mm)	Length (mm)	Width (mm)	Overlap Region (mm)
0.5	76	16	16
1	100	25	25

**Table 2:** Mechanical properties of AISI 304 stainless steel.

Tensile Strength (MPa)	Yield Strength (MPa)	Poisson’s Ratio	Young’s Modulus (GPa)	Elongation (%)
505	215	0.29	193	40

**Table 3:** Chemical composition of AISI 304 stainless steel.

Element	C	Mn	P	S	Si	Cr	Ni	Cu	Mo	Fe
wt.%	0.08	2.0	0.04	0.03	1.0	18	8	0.75	0.75	Balance

**Table 4:** RSW experimental parameters according to the design of the experiment (DOE).

Trial No.	Welding Cases			Welding Current (A)	Pressure (bar)	Welding Time (s)	Squeeze Time (s)	Holding Time (s)	Pulse Welding (−)
1	D1	G1	H1	5000	2.0	0.6	0.6	0.50	1
2	D2	G2	H2	5000	3.5	0.8	0.8	0.75	2
3	D3	G3	H3	5000	5.0	1.0	1	1.00	3
4	D4	G4	H4	5000	6.5	1.2	1.2	1.25	4
5	D5	G5	H5	5000	8.0	1.4	1.4	1.50	5
6	D6	G6	H6	5500	2.0	0.8	1.0	1.25	5
7	D7	G7	H7	5500	3.5	1.0	1.2	1.50	1
8	D8	G8	H8	5500	5.0	1.2	1.4	0.50	2
9	D9	G9	H9	5500	6.5	1.4	0.6	0.75	3
10	D10	G10	H10	5500	8.0	0.6	0.8	1.00	4
11	D11	G11	H11	6000	2.0	1.0	1.4	0.75	4
12	D12	G12	H12	6000	3.5	1.2	0.6	1.00	5
13	D13	G13	H13	6000	5.0	1.4	0.8	1.25	1
14	D14	G14	H14	6000	6.5	0.6	1.0	1.50	2
15	D15	G15	H15	6000	8.0	0.8	1.2	0.50	3
16	D16	G16	H16	6500	2.0	1.2	0.8	1.50	3
17	D17	G17	H17	6500	3.5	1.4	1.0	0.50	4
18	D18	G18	H18	6500	5.0	0.6	1.2	0.75	5
19	D19	G19	H19	6500	6.5	0.8	1.4	1.00	1
20	D20	G20	H20	6500	8.0	1.0	0.6	1.25	2
21	D21	G21	H21	7000	2.0	1.4	1.2	1.00	2
22	D22	G22	H22	7000	3.5	0.6	1.4	1.25	3
23	D23	G23	H23	7000	5.0	0.8	0.6	1.50	4
24	D24	G24	H24	7000	6.5	1.0	0.8	0.50	5
25	D25	G25	H25	7000	8.0	1.2	1.0	0.75	1

Where case D—AISI 304 with a similar thickness of 1 mm, case G—AISI 304 with a similar thickness of 0.5 mm, case H—AISI 304 with dissimilar thicknesses of 1 and 0.5 mm.

**Table 5:** Measured nugget diameter.

Sample No.	Welding Case		
	D	G	H
1	Interfacial	3.2	4.1
2	4.1	3.5	5
3	4.3	3.9	4.2
4	5.2	3.4	4.8
5	4.2	3.8	3.8
6	Partial	3.7	3.1
7	3.9	2.5	3.7
8	5.6	3.2	3.7
9	4.5	3.6	3.5
10	4.9	2.9	3.9
11	5.4	3.4	4.6
12	5.1	3.3	3.2
13	5.1	3.2	3.5
14	5.3	3.4	4.4
15	Interfacial	3.4	3.7
16	5.3	4.1	4.5
17	5.4	3.3	3
18	4.8	3.6	4.4
19	Interfacial	3	2.9
20	4.2	4.2	4.7
21	4.1	4.4	5.5
22	4.6	2.9	4.8
23	4.6	3.9	4.9
24	4.4	4.1	4.4
25	5.3	3.3	4.8

**Table 6:** Validation metrics for analyzing the shear force of the AISI 304 welded joints with the one-output neural network structure.

Training Function	Transfer Function	MSE	R^2^
Trainlm	Logsig	0.01908	0.99788
	Tansig	0.05653	0.99407
	Purelin	0.26678	0.97076
Trainscg	Logsig	0.04536	0.99514
	Tansig	0.05289	0.99416
	Purelin	0.29513	0.96714
Traincgf	Logsig	0.07000	0.99222
	Tansig	0.07847	0.99144
	Purelin	0.25318	0.97228
Traincgb	Logsig	0.04119	0.99567
	Tansig	0.05444	0.99408
	Purelin	0.2519	0.97179
Traincgp	Logsig	0.05179	0.99424
	Tansig	0.06176	0.99318
	Purelin	0.24972	0.9721
Trainbfg	Logsig	0.03759	0.99592
	Tansig	0.08850	0.99014
	Purelin	0.255	0.97156
Trainrp	Logsig	0.03735	0.99617
	Tansig	0.06900	0.99245
	Purelin	0.25375	0.97185
Trainbr	Logsig	0.28144	0.97185
	Tansig	0.33608	0.9621
	Purelin	0.338416	0.96205
Trainoss	Logsig	0.06980	0.99238
	Tansig	0.09637	0.9894
	Purelin	0.27603	0.97016
Traingd	Logsig	0.15347	0.98327
	Tansig	0.32004	0.97016
	Purelin	0.32920	0.96428
Traingdm	Logsig	0.24612	0.9724
	Tansig	0.32344	0.97004
	Purelin	0.35668	0.96433
Traingda	Logsig	0.06624	0.9927
	Tansig	0.32344	0.97004
	Purelin	0.35668	0.96433
Traingdx	Logsig	0.17380	0.98123
	Tansig	0.26535	0.97032
	Purelin	0.29321	0.96812

**Table 7:** Validation metrics for predicting the accuracy of the nugget diameter in the one-output neural network structure.

Training Function	Transfer Function	MSE	R^2^
Trainlm	Logsig	0.02580	0.99091
	Tansig	0.13031	0.95329
	Purelin	0.83903	0.7047
Trainscg	Logsig	0.17665	0.93578
	Tansig	0.05820	0.98032
	Purelin	0.81019	0.73527
Traincgf	Logsig	0.139132	0.94909
	Tansig	0.18729	0.93096
	Purelin	0.84797	0.72979
Traincgb	Logsig	0.11641	0.95858
	Tansig	0.09590	0.96821
	Purelin	0.72475	0.8113
Traincgp	Logsig	0.19795	0.92852
	Tansig	0.21466	0.92476
	Purelin	0.82022	0.73566
Trainbfg	Logsig	0.03759	0.95135
	Tansig	0.10640	0.96223
	Purelin	0.86485	0.72187
Trainrp	Logsig	0.05542	0.98073
	Tansig	0.10463	0.96392
	Purelin	0.84829	0.71437
Trainbr	Logsig	0.29733	0.70413
	Tansig	0.301	0.69975
	Purelin	0.30567	0.69408
Trainoss	Logsig	0.10260	0.96309
	Tansig	0.11626	0.95817
	Purelin	0.81678	0.73098
Traingd	Logsig	0.24465	0.91086
	Tansig	0.27433	0.89832
	Purelin	1.01768	0.65619
Traingdm	Logsig	0.19487	0.92905
	Tansig	0.19829	0.92759
	Purelin	0.84687	0.71004
Traingda	Logsig	0.22783	0.91632
	Tansig	0.18138	0.93427
	Purelin	0.84176	0.70133
Traingdx	Logsig	0.19044	0.93128
	Tansig	0.22155	0.92031
	Purelin	0.81272	0.72265

**Table 8:** Validation metrics for predicting the shear force and nugget diameter in the two-output neural network structure.

Training Function	Transfer Function	MSE	R^2^
Trainlm	Logsig	0.05172	0.99183
	Tansig	0.17230	0.97331
	Purelin	0.55255	0.91556
Trainscg	Logsig	0.26193	0.95646
	Tansig	0.32907	0.94515
	Purelin	0.63906	0.8975
Traincgf	Logsig	0.22655	0.96265
	Tansig	0.32250	0.94737
	Purelin	0.57183	0.90784
Traincgb	Logsig	0.23511	0.95952
	Tansig	0.32801	0.94826
	Purelin	0.7893	0.88304
Traincgp	Logsig	0.20792	0.96757
	Tansig	0.29317	0.95412
	Purelin	0.73110	0.88127
Trainbfg	Logsig	0.23088	0.96155
	Tansig	0.40197	0.93619
	Purelin	0.89357	0.87887
Trainrp	Logsig	0.38726	0.93603
	Tansig	0.49935	0.91774
	Purelin	0.84987	0.86307
Trainbr	Logsig	0.34984	0.94128
	Tansig	0.49236	0.91857
	Purelin	0.86289	0.84892
Trainoss	Logsig	0.16953	0.97305
	Tansig	0.26288	0.95616
	Purelin	0.79617	0.86795
Traingd	Logsig	0.70485	0.88168
	Tansig	0.71660	0.87863
	Purelin	1.0844	0.80303
Traingdm	Logsig	0.76383	0.86808
	Tansig	0.92812	0.85712
	Purelin	1.1785	0.7926
Traingda	Logsig	0.54909	0.90771
	Tansig	0.82539	0.86649
	Purelin	0.90740	0.85849
Traingdx	Logsig	0.47430	0.92043
	Tansig	0.58618	0.91149
	Purelin	0.82792	0.86647

**Table 9:** Evaluation of the best ANN model based on the validation metrics.

One-Output Structure with Trainlm and Logsig						
Validation Metrics	MSE	R^2^	ME	MAE	RMSE	MRE
Shear force	0.01908	0.99788	−0.00187	0.08953	0.13813	5.55869 × 10^−6^
Nugget diameter	0.02580	0.99091	−0.01059	0.10396	0.16063	3.6445 × 10^−5^
Two-output structure with Trainlm and Logsig						
Shear force and nugget diameter	0.05172	0.99183	−0.0343	0.15726	0.22743	5.4585 × 10^−5^

**Table 10:** Weights and biases for predicting shear force with Trainlm and Logsig.

b1					b2				
6.0593					1.3804				
−4.3523					−0.18468				
−0.57682					1.109				
−1.9088									
2.0805									
2.6624									
1.125									
3.0614									
−5.0681									
−2.5899									
IW									
−2.0687		2.9401	−2.168		0.0038676		−0.81016		0.81156
0.75353		0.034347	1.2038		0.15507		−3.4153		2.1785
−1.1868		−2.3461	−2.8087		−0.38282		2.2392		3.7214
3.3961		1.6705	−1.066		−1.3043		−2.4845		3.8159
−1.8535		−5.0655	−5.5748		0.09913		1.2594		−1.298
−0.038716		−6.0105	−0.96251		−0.4567		−2.7778		1.6609
−0.78585		−0.94715	1.422		−2.2769		1.5962		2.8447
1.9345		1.7366	−3.5865		0.89875		−1.5641		−1.5636
0.60689		2.843	0.92038		2.6216		−0.2828		0.5321
0.05569		−1.3053	3.5661		1.3969		−3.3934		−0.94312
LW									
−1.6326	1.1531	3.4298	−0.78814	−2.1214	−1.2433	0.050153	0.78538	−3.7281	2.0756
−2.129	0.31974	1.5952	−1.5102	−3.2758	1.071	1.3947	3.3428	1.1558	−1.0281
−2.805	0.25463	−2.0404	−0.98168	−3.1905	1.194	0.34219	2.6929	−0.72115	−0.37251

**Table 11:** Weights and biases for predicting nugget diameter with Trainlm and Logsig.

b1					b2				
4.4415					−0.23673				
2.0865					0.82771				
3.0533					−0.35106				
−2.4003									
2.2273									
0.75977									
3.6923									
−0.97346									
−0.64778									
−5.7068									
IW									
2.6346		−0.53884	−3.5953		4.3189		2.0695		−1.6533
−4.214		3.6914	0.55714		6.6269		2.1891		−1.3247
−1.0584		−3.0337	1.6359		−5.4911		−1.8593		2.7159
−2.5806		−4.2608	1.3269		3.0904		0.055187		−2.3917
−7.7207		1.8558	1.5305		2.3453		−4.317		0.21968
3.0862		3.4386	−3.5603		−0.19471		2.4631		−2.9977
7.6645		−4.6628	3.1247		1.0516		1.2497		−3.3013
2.9967		2.6921	2.5192		0.20438		−1.2481		4.1015
4.49		1.6454	−0.17563		3.4924		1.272		−1.2577
−1.5083		0.88408	1.1391		0.71768		−1.9169		−0.56682
LW									
−5.052	9.7582	−0.87758	0.75098	−8.3978	0.25209	4.3682	2.2562	−6.113	1.1958
−1.2057	−0.15235	1.4335	−1.6726	−0.46344	−1.8993	−0.96709	−1.3038	3.2362	−0.95902
0.1269	1.3874	1.0573	2.3768	−3.2144	0.37308	−1.5729	1.3968	0.14822	0.047946

## Data Availability

Data are contained within the article.
